# Statistical methods for constructing disease comorbidity networks from longitudinal inpatient data

**DOI:** 10.1007/s41109-018-0101-4

**Published:** 2018-11-07

**Authors:** Babak Fotouhi, Naghmeh Momeni, Maria A. Riolo, David L. Buckeridge

**Affiliations:** 1 Program for Evolutionary Dynamics, Harvard University, Cambridge, USA; 2 Sloan School of Management, Massachusetts Institute of Technology, Cambridge, USA; 3 Center for the Study of Complex Systems, University of Michigan, Ann Arbor, Michigan USA; 4 Department of Epidemiology, Biostatistics, and Occupational Health, McGill University, Montreal, Canada

**Keywords:** Weighted networks, Null model, Comorbidity, Disease networks, Centrality

## Abstract

Tools from network science can be utilized to study relations between diseases. Different studies focus on different types of inter-disease linkages. One of them is the comorbidity patterns derived from large-scale longitudinal data of hospital discharge records. Researchers seek to describe comorbidity relations as a network to characterize pathways of disease progressions and to predict future risks. The first step in such studies is the construction of the network itself, which subsequent analyses rest upon. There are different ways to build such a network. In this paper, we provide an overview of several existing statistical approaches in network science applicable to weighted directed networks. We discuss the differences between the null models that these models assume and their applications. We apply these methods to the inpatient data of approximately one million people, spanning approximately 17 years, pertaining to the Montreal Census Metropolitan Area. We discuss the differences in the structure of the networks built by different methods, and different features of the comorbidity relations that they extract. We also present several example applications of these methods.

## Introduction

In the last decade, several network approaches have been introduced to study the interrelations between human diseases. Networks are constructed by connecting diseases that share certain features, collapsing a bipartite graph into a unipartite graph. Examples include genetic/interactomic association (Goh et al. 2007; Halu et al. 2017; Menche et al. 2015), similarity of symptoms (Zhou et al. 2014; Halu et al. 2017), similarity of pertinent drugs (Yıldırım et al. 2007), commonality of etiological environmental factors associated with diseases (Liu et al. 2009), adjacency of metabolic reactions catalyzed by corresponding mutated enzymes (Lee et al. 2008), and co-occurrence in patients (Hidalgo et al. 2009; Folino et al. 2010; Chmiel et al. 2014; Jensen et al. 2014; Jeong et al. 2017). Also sometimes more than one of these networks are juxtaposed to build a multiplex characterization (Halu et al. 2017). All of these strands of research are beneficial and insight-engendering in their respective contexts, and the increase in the breadth of topics and the diversity of approaches promises the emergence of a new field of research.

Here we focus on a methodological problem in this new field. We investigate different statistical methods for defining a weighted and directed co-morbidity network from longitudinal hospital in-patient data, and show that different methods capture different aspects of co-morbidity relations. We use a data set containing over a million people for a period of approximately 17 years, and employ different statistical methods to extract co-morbidity networks based on this data set.

Some of the previous studies have used a binary version of the comorbidity networks to study the structural properties of diseases (Hidalgo et al. 2009; Folino et al. 2010; Chmiel et al. 2014; Jeong et al. 2017). Measures for establishing unweighted binary links between disease pairs include the *ϕ*-correlation (which is closely linked to the *χ*^2^ statistic) and relative risk (ratio of observed co-occurrence of a pair to the expected co-occurrence of a null model) (Hidalgo et al. 2009; Folino et al. 2010; Chmiel et al. 2014; Jeong et al. 2017). These methods capture useful information about co-morbidities, and also have drawbacks. The *ϕ*-correlation underestimates the associations in disease pairs in which one disease is rare and the other is prevalent. The relative risk tends to overestimate linkages between rare diseases and to underestimate those between prevalent diseases. To use any of these methods, one inevitably chooses trade-off parameters to construct the network with reasonable accuracy. Examples include the thresholds in Ref. Chmiel et al. (2014), the choice of relative risk cutoff (4 in Ref. Jeong et al. (2017) and 20 in Ref. Folino et al. (2010)), and the choice of defining “lop-sided”ness if one direction of a reciprocal link weights at least twice as the other direction (Jeong et al. 2017). These thresholds are chosen to be intuitively-reasonable values considering the respective settings.

In this paper, we study different systematic statistical methods for building weighted directed comorbidity networks. These methods use different criteria to deem statistical significance for links. The resulting networks are sparser than the raw network, and the links are in some sense adjudicated as meaningful, that is, non-noise. In addition to statistical considerations, working with sparser networks is easier both computationally and intuitively, and the ultimate goal of gaining insight about paths of disease progression is facilitated. Here we investigate the effect of the statistical procedure used to build a network from the disease co-occurrence data on the structure of the resulting network. We show that depending on the null model used for defining the statistical significance of disease-disease links, different aspects of the comorbidity patterns are captured, and the resulting networks can have different micro/meso structures, and the centrality/ranking measures of individual diseases can differ. We describe the networks built from each method, discuss their similarities and differences, and present several example applications using these constructed networks.

## Data

Using the registry of all medically insured people in the province of Québec (fichier d’inscription des personnes assures - FIPA) we randomly sampled 25% of the people residing in the Montreal Census Metropolitan Area (CMA) in 1998. In each subsequent year, we used the FIPA to re-sample immigrants to the CMA and babies born to mothers residing in the CMA to maintain a representative, 25% sample for each year. For sampled individuals, we obtain regular data updates from the Régie de l’assurance maladie du Québec (RAMQ) on physician billing, drugs dispensed, hospitalization records, and death certificates. The data sets are linked with an anonymized unique identifier. At any given time, the dynamic cohort contains approximately 1 million people and follow-up data span approximately 17 years.

Moreover, in one of the applications that we present below, we use the dataset that is publicly available via Ref. Park et al. (2009) to connect our results to previous findings in the literature. In this data set, the protein–protein interaction (PPI) and coexpression networks and the inter-disease network of shared genes are linked to the comorbidity network derived from US Medicare claims of over 13 million elderly patients. The data set can be accessed online via http://msb.embopress.org/content/5/1/262.

The analyses reported in this paper has been conducted using MATLAB R2015b.

## Network construction methods

### ICD codes

We use the ICD9 coding scheme for the classification of diseases. To make the analysis more tractable, we confine the analysis to the 3-digit classification.

### Network terminology

Throughout, the pathways of disease progression are modeled by a network, where nodes represent diseases and a link from node *i* to node *j* represents an instance of diagnosis of disease *i* followed by a subsequent diagnosis of disease *j*. We denote the number of connections of a node by its *degree*, denoted by *k*. The weight of the link from disease *i* to disease *j* is denoted by *w*_*ij*_, which is equal to the number of times a diagnosis of disease *i* followed by a diagnosis of disease *j* is reported in the data set. By the *strength* (Serrano et al. 2009) of a node, denoted by *s*, we refer to the sum of the weights of its links. We use these for either directions of the links. For example, the ‘out-strength’ $s_{x}^{\text {out}}={\sum \nolimits }_{y} w_{xy}$ denotes the sum of the weights of the out-links of node *x* to other nodes, and the out-degree $k_{x}^{\text {out}}$ denotes the number of such out-links. The out-strength of a node is equal to the total number of times that the diagnosis of that disease was followed by the diagnosis of any other disease. The out-degree of a node is the number of distinct diseases that follow that particular disease, without counting the multiplicities. Similarly we can define the in-strength and in-degree for each node. We denote the sum of the strength of all links by *S*, that is, we have $S={\sum \nolimits }_{ij} w_{ij}$.

### Raw network

**Fig. 1:**
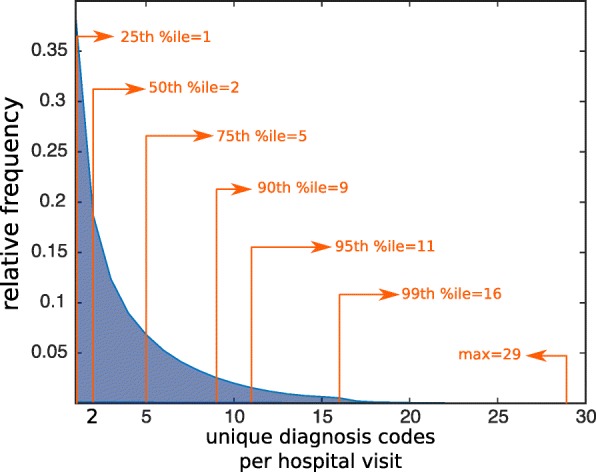
Hospital visits. The distribution of the number of ICD9-coded diagnosis per hospital visit

**Fig. 2:**
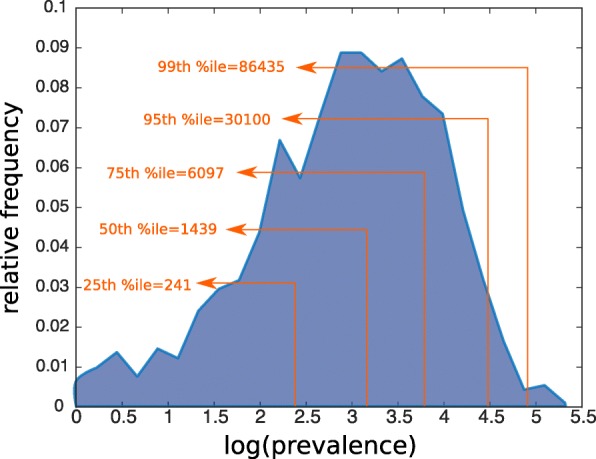
Distribution of disease prevalence. The logarithm of the prevalences is normal-like, but the Kolmogorov-Smirnov test rejects the normality hypothesis

In our data set, there are 1,700,000 distinct hospital visits, and the total number of unique ICD9-coded diagnoses is 6,500,000. Among all the hospital visits, 35.3*%* where given only one ICD9-coded diagnosis. Figure 1 presents the histogram of the number of ICD9-coded diagnoses per hospital visit. Table 1 presents the top 10 disease in the data set with highest prevalence. Figure 2 depicts the histogram of the prevalence of the diseases in our data set. The distribution of the logarithm of the prevalences is normal-like, but the result of the Kolmogorov-Smirnov test was that the normality assumption is rejected (on the 0.1 level). Though not strictly log-normal, the prevalence distribution is evidently heavy-tailed, that is, most diseases have low levels of prevalence and a minority of the diseases have extremely high levels of prevalence. The starting point of our analysis is to build a raw network, which will be the substrate on which other methods construct different derived networks.

**Table 1:** Top 10 most-prevalent diseases in our data set

Rank	ICD-9	Disease description	Prevalence (× 1000)
1	250	Diabetes mellitus	206
2	414	Chronic ischemic heart disease	205
3	272	Disorders of lipoid metabolism	198
4	366	Cataract	154
5	427	Cardiac dysrhythmias	148
6	401	Essential hypertension	141
7	244	Acquired hypothyroidism	110
8	285	Other and unspecified anemias	107
9	041	Bacterial infection [unspecified site]	95
10	664	Trauma to perineum and vulva during delivery	80

**Fig. 3:**
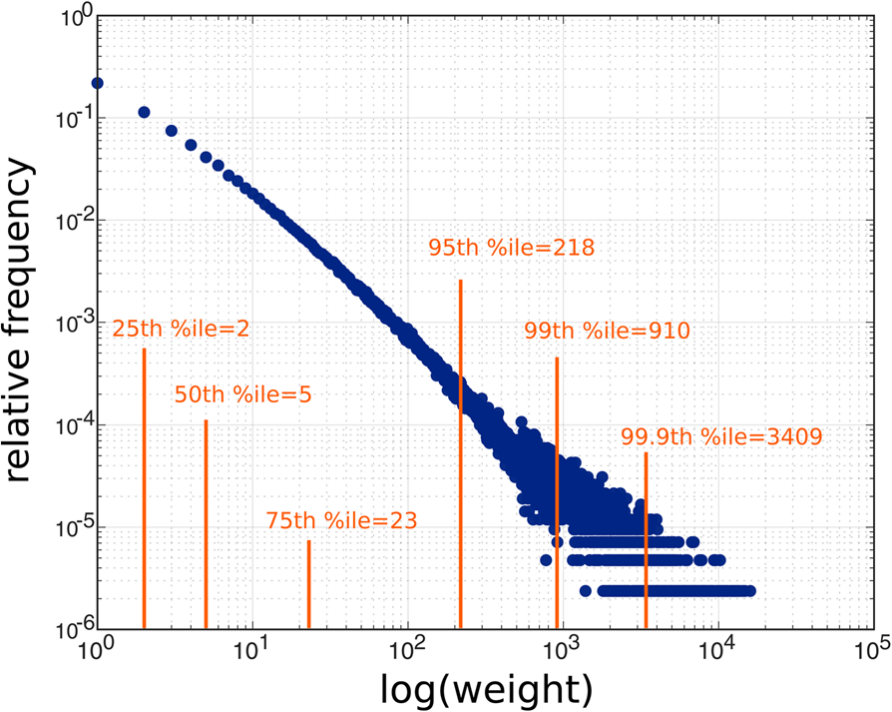
Distribution of link weights. The distribution of link weights. According to the method devised in Ref. Clauset et al. (2009), the null hypothesis that the weights have a power-law distribution is rejected

We seek a weighted and directed characterization of the comorbidity patterns, where the weight of the link from disease *i* to *j* equals the number of instances where a patient with disease *i* later developed disease *j*. The raw network is made by sweeping over every hospital visit, and incrementing the weight of the link from disease *i* to disease *j* if in that visit disease *j* is diagnosed for the first time in a patient for whom disease *i* had been previously diagnosed. In other words, if a patient who previously had disease *i* but did not have disease *j* visits the hospital and is diagnosed with disease *j*, then *w*_*ij*_ increments by one. There are hospital visits where two diseases are co-diagnosed in a patient for the first time. We do not observe what the temporal order of their occurrence was before the patient visited the hospital. The link between the two diseases might be in either direction. We have two possible choices: either to discard this observation or to count this link in both directions. We choose the former, because we prefer less data to more-but-noisy data. About 35% of the patients in the data set only visited the hospital once, thus they did not constitute any comorbidity trajectory with the above criteria, and did not contribute to the raw network. The weight distribution of the links are depicted in Fig. 3. After undertaking the maximum likelihood method devised in Clauset et al. (2009), we conclude that although the distribution resembles a linear curve on the log-log scale, the hypothesis that the weight distribution is power-law is rejected. Thus throughout the paper we only use non-parametric statistical methods. We do not assume scale-freeness of the distributions.

### Relative risk and observed-to-expected ratio

The relative risk (*RR*) is a measure of comorbidity strength used in previous studies of disease networks (Hidalgo et al. 2009; Park et al. 2009; Jeong et al. 2017). In these studies, the relative risk is equal to the ratio of the number of times that an ordered pair of diseases occur in the empirical data to the expected number of times it would occur in a random network. In Ref. Hidalgo et al. (2009), the threshold value for this quantity was obtained from formulas for confidence intervals given in Ref. Katz et al. (1978).

Here we make a notational clarification. The problem considered in Ref. Katz et al. (1978) is the problem of finding confidence intervals for risk ratios in the following sense. Consider the 2×2 contingency table given in Table 2. In the following calculations, we use the values of *a*,*b*,*c*,*d* defined in Table 2 for brevity of notation.

**Table 2:** The contingency table to analyze the comorbidity of diseases *i* and *j*

	Second disease is *j*	Second disease is not *j*	Total
First disease is *i*	*a*=*w*_*ij*_/*S*	$b=s_{i}^{\text {out}}/S -w_{ij}/S$	$a+b=s_{i}^{\text {out}}/S $
First disease is not *i*	$c=s_{j}^{\text {in}}/S-w_{ij}/S $	$d=1-s_{i}^{\text {out}}/S -s_{j}^{\text {in}}/S + w_{ij}/S$	$c+d=1-s_{i}^{\text {out}} /S$
Total	$a+c=s_{j}^{\text {in}}/S $	$b+d=1 -s_{j}^{\text {in}}/S $	1

The sum of all link weights in the network is denoted by *S*

The relative risk considered in Ref. Katz et al. (1978), and conventionally in epidemiology and biostatistics, is defined as ${\left (\frac {a}{a+b}\right)/ \left (\frac {c}{c+d}\right)}$. But that is not how *RR* is defined in Refs. Hidalgo et al. (2009); Jeong et al. (2017); Park et al. (2009) to study comorbidity links. Rather, these studies define *RR* as ${\frac {a }{(a+b)(a+c)}}$. The numerator is the observed co-occurrence proportion, and the denominator is the expected co-occurrence proportion under independence. The quantity ${\frac {a}{(a+b)(a+c)}}$ is actually what is often called the *observed-to-expected ratio*. Herein we denote it by *OER*. So we use this terminology in our paper:

1 $$\begin{array}{*{20}l} { OER_{ij}= \frac{ w_{ij}\times S}{ s_{j}^{\text{in}} s_{i}^{\text{out}}}. }\end{array} $$

Note that we can equivalently write:

2 $$\begin{array}{*{20}l} { \log OER_{ij} = \log \frac{\frac{w_{ij}}{S}}{\left(\frac{s_{j}^{\text{in} }}{S} \right) \times\left(\frac{s_{i}^{\text{out}}}{S} \right)}. }\end{array} $$

In this form, log*OER* is equivalent to the point-wise mutual information that is used, for example, in natural language processing to measure how likely two words are to co-occur (Bouma 2009). The confidence intervals for *OER* can be obtained by applying the delta method to log*OER*:

3 $$\begin{array}{*{20}l} \text{var}(\log OER) &= \frac{1}{S} \left[ \begin{array}{c} \frac{b c-a^{2}}{a (a+b) (a+c)} \\ \frac{-1}{ a + b} \\ \frac{ -1 }{ a+c} \\ 0 \end{array} \right]^{T} \left[ \begin{array}{cccc} a(1-a) & - a b & - a c & - a d \\ - a b & b(1-b) & - b c & - b d \\ - a c & - b c & c(1-c) & - c d \\ - a d & - b d & - c d & d(1-d) \end{array} \right] \left[ \begin{array}{c} \frac{b c-a^{2}}{a (a+b) (a+c)} \\ \frac{-1}{ a + b} \\ \frac{ -1 }{ a+c} \\ 0 \end{array} \right] \\ &= \frac{ bc(1-a)+a^{2}(1-b-c)-a^{3}}{a (a+b) (a+c)S},\end{array} $$

where *T* denotes transpose. So the 95% confidence intervals are obtained as follows: $ \text {CI}(OER)= \left [\widehat {OER} e^{-1.96 \sqrt {\text {var}(\log OER)}}~,~ \widehat {OER} e^{+1.96 \sqrt {\text {var}(\log OER)}} \right ] $.

We re- iterate that what we have defined here as *OER* is what the previous studies of comorbidity networks have referred to as the relative risk, and what we define below as the relative risk is not to be confused with their notation. As discussed above, for the relative risk we define:

4 $$\begin{array}{*{20}l} { RR= \frac{\frac{w_{ij}}{s_{i}^{\text{out}}}}{\frac{s_{j}^{\text{in}}-w_{ij}}{S-s_{i}^{\text{out}}}} }\end{array} $$

So the relative risk is the ratio of the probability that *j* receives one of the out-links of *i* to the probability that *j* receives a link from another disease that is not *i*. In other words, the relative risk is the ratio of the probability that disease *j* succeeds disease *i* to the probability that disease *j* succeeds any other disease. For the variance of the relative risk, we can proceed similar to before and obtain the following result which is equivalent to what is used in Ref. Hidalgo et al. (2009) for *OER*:

5 $$\begin{array}{*{20}l} {}\text{var}(\log RR) &= \frac{1}{S}{ \left[ \begin{array}{c} \frac{ b }{ a (a + b)} \\ \frac{-1}{ a + b} \\ \frac{ -d }{ c(c+d)} \\ \frac{1}{ c + d } \end{array} \right]^{T} \left[ \begin{array}{cccc} a(1-a) & - a b & - a c & - a d \\ - a b & b(1-b) & - b c & - b d \\ - a c & - b c & c(1-c) & - c d \\ - a d & - b d & - c d & d(1-d) \end{array} \right] \left[ \begin{array}{c} \frac{ b }{ a (a + b)} \\ \frac{-1}{ a + b} \\ \frac{ -d }{ c(c+d)} \\ \frac{1}{ c + d } \end{array} \right]}\\ &=\frac{ b}{ a (a+b)S} + \frac{ d}{ c(c+d)S} \end{array} $$

Despite the technical distinction between *RR* and *OER*, we can show that for practical purposes considered in this paper, these two measures are very close for almost all cases. Dividing Eq. 1 by Eq. 4, we have:

6 $$\begin{array}{*{20}l}{ \frac{RR}{OER}= \frac{1-w_{ij}/s_{j}^{\text{in}}}{1-s_{i}^{\text{out}}/S} \simeq 1-w_{ij}/s_{j}^{\text{in}}. }\end{array} $$

This ratio is very close to one if *w*_*ij*_ is much smaller than $s_{j}^{\text {in}}$, that is, if disease *i* is not a main predecessor of disease *j* in comorbidity patterns. However, if the in-degree of disease *j* is small, so that it has few predecessors, then *RR* might deviate from *OER*. In our data set, there are only 61 disease pairs for which $w_{ij}/s_{j}^{\text {in}}$ exceeded 10%. Since this fraction is negligibly small, the *RR* and *OER* measures are therefore almost identical. We use the network constructed based on *OER* in the following analyses to be consistent with the measures used in the previous literature.

A practical caveat of OER is that diseases with very low prevalence can produce unduly large values of OER, which is evident from Eq. 2. This is also pointed out previously in the disease networks literature (Park et al. 2009). A workaround is to discard disease pairs for which the expected co-occurrence under independence is greater than a certain threshold. As investigated in Ref. Park et al. (2009), as long as the threshold exceeds unity, the structure of the OER comorbidity network remains robust against the choice of threshold. In the present paper, we choose the threshold to be equal to unity.

### *ϕ* coefficient

The *ϕ* correlation coefficient is a measure of association for two binary variables (here, the binary variable indicates whether or not a certain disease is diagnosed). It quantifies the tendency of the two binary variables to co-occur, that is, the concentration of the contingency table towards the diagonal. Generalizing the undirected case considered before in the literature (Hidalgo et al. 2009), we can define the directed version of the *ϕ* coefficient as follows:

7 $$\begin{array}{*{20}l}{ \phi_{ij}= \frac{w_{ij} S- s_{i}^{\text{out}} s_{j}^{\text{in}}} {\sqrt{ s_{i}^{\text{out}} s_{j}^{\text{in}} \left(S- s_{i}^{\text{out}}\right) \left(S- s_{j}^{\text{in}}\right) }}.}\end{array} $$

If the instances of disease *j* succeeding disease *i* are more frequent than the random case, the *ϕ* coefficient will be positive. If these instances are less frequent than it would be expected for the random case, the *ϕ* coefficient will be negative, and it means that having developed disease *i* actually decreases getting disease *j*. The caveat in the performance of the *ϕ* coefficient is that, the higher the disparity between the prevalences of the two diseases, the less informative the *ϕ* coefficient becomes (Hidalgo et al. 2009). A technical caveat of the *ϕ* coefficient is that, although it is always in the [−1,+1] interval, the absolute values of its theoretical extrema are less than unity (Guilford 1965; Davenport Jr and El-Sanhurry 1991; Park et al. 2009). In the case of positive association, assuming $s_{j}^{\text {in}}< s_{i}^{\text {out}}$, the maximum value of *ϕ* which is theoretically attainable is $\phi _{\text {max}}=\frac {\left (S-s_{i}^{\text {out}}\right) s_{j}^{\text {in}}}{\left (S-s_{j}^{\text {in}}\right)s_{i}^{\text {out}}}$. Because both $s_{i}^{\text {out}}$ and $s_{j}^{\text {in}}$ are much smaller than *S* in our analysis, this maximum value is close to $s_{j}^{\text {in}}/s_{i}^{\text {out}}$, which is less than unity. If we had started with the assumption $s_{j}^{\text {in}}>s_{i}^{\text {out}}$, we would have found the inverse of the latter fraction, so the maximum value would again be smaller than unity. A possible workaround would be to normalize *ϕ* via dividing it by *ϕ*_max_ (Davenport Jr and El-Sanhurry 1991; Park et al. 2009; Chmiel et al. 2014).

The *ϕ* coefficient is related to the conventional *χ*^2^ statistic in the following way: *ϕ*^2^=*χ*^2^/*S*. To determine statistical significance and to find a p-value for the *ϕ* coefficient, Ref. Hidalgo et al. (2009) has employed a t-distribution approximation. The conditions for the validity of the approximation are not discussed. Ref. Chmiel et al. (2014) uses a *χ*^2^ test. Moreover, it is important to note that the *χ*^2^ test involves underlying assumptions regarding minimum expected cell counts in the 2×2 table (Everitt 1992). In our setting, characterized by Table 2, the expected cell count for the (1,1) cell is $s_{i}^{\text {out}}s_{j}^{\text {in}}/S$, which is smaller than unity for many existing disease pairs in our data set. Thus the requirements for the *χ*^2^ test are not always met. In Ref. Park et al. (2009), the distributions of *w*_*ij*_ is assumed to be binomial, and a Poisson approximation is used to calculate the p-values. It is of note that the Poisson approximation to the binomial distribution also requires the expected co-occurrence of the disease pairs not to be large (usually *O*(1) is advised in statistics textbooks). For about 10% of the disease pairs in our data set, this condition is not met. So this method is also not applicable to our data set. In this paper, we used Fisher’s exact test to decide the significance of association.

### Disparity filter

The disparity filter (DF) has a local node-based approach to define the network null model (Serrano et al. 2009). We first consider unweighted networks for explanatory purposes. The DF method asks that, for node *x* with given strength *s*_*x*_ and degree *k*_*x*_, what would we expect the weights of its links to look like if they were allocated randomly? In the null model of the DF method, each node is assumed to possess a given strength *s*_*x*_ that is to be distributed among its *k*_*x*_ neighbors uniformly at random. That is, if the null is true, node *x* has no preference among its neighbors, and it would distribute its weights uniformly at random. We can mathematically conceptualize this setting as follows: In the unit interval [0,1], *k*−1 points are drawn uniformly at random, thus leading to *k* shares of strength pertaining to *k* different links. Without loss of generality, consider the left-most interval (between 0 and the left-most randomly-chosen point). The probability that the length of this share (which corresponds to the weight of one of the links) is greater than *x* is equal to the probability that all of the other *k*−1 points fall in a piece with length 1−*x*. This probability equals (1−*x*)^*k*−1^. This coincides with the *p*-value for that link, because it gives the probability that, under the null, the share of that link exceeds the observed value.

The said procedure can be undertaken either for out-degrees or in-degrees, yielding two distinct backbone networks. We define the *p*-value:

8 $${ \left\{\begin{array}{l} \alpha_{ij}^{\text{out}}= \left(1-\frac{w_{ij}}{s_{i}^{\text{out}}}\right)^{\left(k_{i}^{\text{out}}-1\right)} \\ \\ \alpha_{ij}^{\text{in}}= \left(1-\frac{w_{ji}}{s_{i}^{\text{in}}}\right)^{\left(k_{i}^{\text{in}}-1\right)} \end{array}\right. } $$

Then, a global thresholding can be done for a desired level of significance by discarding links whose *α* values exceed a certain threshold value. In this paper we set *α*=0.05 as the threshold value. We only consider the out-network in this study, because we are concerned mainly with finding diseases that increase the risk of developing other diseases (that is, those who perform as ‘roots’ in comorbidity paths).

### Iterative proportional fitting procedure

The Iterative Proportional Fitting Procedure (IPFP) is a simple method used in the context of US inter-county migration flows (Slater 2009a; 2009b). Here we utilize it to analyze disease flows. This method utilizes the Sinkhorn-Knopp algorithm (Sinkhorn and Knopp 1967), which involves iteratively normalizing the rows and columns of the adjacency matrix until the row and column sums are sufficiently close to unity. In the IPFP method, after constructing this bistochastic matrix via successive normalizations, we start from an empty network and add the links successively in the decreasing order of their weight in the bistochastic matrix until the largest connected component of the network comprises every node. One can also use a global thresholding to obtain sparser networks. As the threshold is lowered, more and more links whose values in the bistochastic matrix exceed the threshold are allowed in. In this paper, we retain the top 5% of the heaviest links of the resultant bistochastic matrix.

The mathematical procedure for the IPFP method is as follows. Suppose we seek *B*, a transformation of the adjacency matrix *A* between the *N* distinct diseases, and we impose the condition that every disease in *B* must have the same number of preceding diagnoses and the same number of succeeding diagnoses as every other disease. If we interpret disease progressions as *flows* between diseases, this condition is the equality of in-flow and out-flow for every disease. This fixed amount is arbitrary and can be set to unity, so the elements of *B* can be interpreted as probabilities. We can also normalize the elements of *A* by *S*, that is, we can interpret *w*_*ij*_/*S* as the fraction of the total inter-disease flux that flows from disease *i* to disease *j*, so it can be interpreted as a probability. Consider the following minimum-cross-entropy estimation problem:

9 $$\begin{array}{*{20}l} \underset{\{B_{ij}\}}{\text{minimize}} \quad &\sum\limits_{i,j=1}^{N} B_{ij} \log \frac{SB_{ij}}{w_{ij}}  \\ \text{subject to} \quad &\sum\limits_{j=1}^{N} B_{ij}=1, ~~~ i=1\ldots N,  \\ &\sum\limits_{i=1}^{N} B_{ij}=1, ~~~ j=1 \ldots N.\end{array} $$

The task is to minimize the Kullback-Leibler divergence between the target matrix *B* and the adjacency matrix *A* (whose *ij* element is *w*_*ij*_), given the normalization constraints of rows and columns of *B*. We denote the Lagrangian multipliers for the first constraint (normalization of rows) by *λ*_*j*_ and those of the second constraint (normalization of columns) by *μ*_*i*_. The Lagrangian is:

10 $$\begin{array}{*{20}l}{ \mathcal{L} = \sum\limits_{ij} B_{ij} \log \frac{SB_{ij}}{w_{ij}} - \sum\limits_{i} \lambda_{i} \left(\sum\limits_{j}B_{ij}-1 \right) - \sum\limits_{j} \mu_{j} \left(\sum\limits_{i} B_{ij}-1 \right).}\end{array} $$

Setting the components of the gradient equal to zero, we get:

11 $$\begin{array}{*{20}l} { \log \frac{SB_{ij}}{w_{ij}} + 1 = \lambda_{i} + \mu_{j} \Longrightarrow B_{ij} =\frac{ w_{ij}}{S} e^{\lambda_{i} + \mu_{j} -1} }\end{array} $$

Let *Λ* be a diagonal matrix with positive elements ${\exp (\lambda _{i}-1/2)/\sqrt {S}}$ and let *M* be a diagonal matrix with positive elements ${\exp (\mu _{j}-1/2)/\sqrt {S}}$. Then, according to Eq. 11, the above-formulated maximum entropy problem becomes equivalent to finding a bistochastic matrix *B* such that: *B*=*ΛAM*, with the following pair of coupled equations holding: $ \Lambda _{ii} = 1/{\sum \nolimits }_{j} A_{ij} M_{jj}$ and $M_{ii}= 1/{\sum \nolimits }_{i} A_{ij} \Lambda _{ii}$. This is equivalent to what the Sinkhorn-Knopp theorem states: iterating the matrices *Λ* and *M* from the latter pair and inserting the limiting result into the equation *B*=*ΛAM*, the unique bistochastic matrix *B* is obtained (Sinkhorn and Knopp 1967). For discussions regarding convergence of the iteration, see Refs. Sinkhorn and Knopp (1967); Chakrabarty and Khanna (2018). In brief, the method converges if and only if the adjacency matrix has total support, which means that for every nonzero element *A*, there exists a column permutation of *A* such that the nonzero element is brought to the main diagonal and every diagonal element is nonzero. Note that adding a nonzero constant to every diagonal element of the adjacency matrix (which is equivalent to adding a self-link for every disease) guarantees this property, because for every off-diagonal element we can simply swap its column such that it is brought to the main diagonal, and the main diagonal of the resulting matrix is already all-positive. Such an addition, akin to Laplace smoothing in machine learning (Schütze et al. 2008), is equivalent to viewing each disease as succeeding itself after a prior diagnosis, because any two checks for the same patient during the period of an illness would produce such a self-link. We performed a robustness check regarding the amount added to the main diagonal. We observed reasonable robustness for any added value up to *O*(10^1^) for the network measures that we invoked in this paper. So we used unity; we increment the diagonal of the adjacency matrix by unity and then applied the IPFP procedure by iteratively normalizing columns and rows until sufficient convergence.

The IPFP method has another property that facilitates the interpretation of its function. Consider the *m*-th stage of the iterative normalization procedure in the IPFP method. Denote the adjacency matrix at this stage by *A*^(*m*)^. Denote the sum of row *i* at this stage by $r_{i}^{(m)}$ and the sum of column *i* by $c_{i}^{(m)}$. Denote the adjacency matrix after row normalization by *A*^(*m*+1/2)^, and denote the result of the subsequent column-normalization by *A*^(*m*+1)^. The element *ij* of *A*^(*m*+1/2)^ is given by

12 $$\begin{array}{*{20}l} { A_{ij}^{(m+1/2)}= \frac{A_{ij}^{(m)}}{\sum\limits_{a} A_{ia}^{(m)}}. }\end{array} $$

After the subsequent column normalization, we have

13 $$\begin{array}{*{20}l}{ A_{ij}^{(m+1)}=\frac{A_{ij}^{(m+1/2)}}{\sum\limits_{b} A_{bj}^{(m+1/2)}}= \frac{\frac{A_{ij}^{(m)}}{\sum\limits_{a} A_{ia}^{(m)}}} {\sum\limits_{b} \frac{A_{bj}^{(m)}}{\sum\limits_{a} A_{ba}^{(m)}}} = \frac{A_{ij}^{(m)}}{ r_{i}^{(m)} \sum\limits_{b} A_{bj}^{(m)}/r_{b}^{(m)}} }\end{array} $$

Therefore, for two disease pairs *ij* and *kℓ*, we have:

14 $$\begin{array}{*{20}l}\frac{A_{ij}^{(m+1)} A_{k\ell}^{(m+1)}}{A_{i\ell}^{(m+1)}A_{kj}^{(m+1)}}= \frac{A_{ij}^{(m)} A_{k\ell}^{(m)}}{A_{i\ell}^{(m)}A_{kj}^{(m)}} \times \left(\frac{r_{i}^{(m)} \sum\limits_{b} A_{b\ell}^{(m)}/r_{b}^{(m)} \times r_{k}^{(m)} \sum\limits_{b} A_{bj}^{(m)}/r_{b}^{(m)}} {r_{i}^{(m)} \sum\limits_{b} A_{bj}^{(m)}/r_{b}^{(m)} \times r_{k}^{(m)} \sum\limits_{b} A_{b\ell}^{(m)}/r_{b}^{(m)}} \right) = \frac{A_{ij}^{(m)} A_{k\ell}^{(m)}}{A_{i\ell}^{(m)}A_{kj}^{(m)}}. \end{array} $$

Thus the quantity $ \left [ A_{ij}^{(m)} A_{k\ell }^{(m)}\right ]/ \left [A_{i\ell }^{(m)}A_{kj}^{(m)}\right ]$ is conserved. This can be interpreted as the odds ratio in the contingency table formed by the four diseases *i*,*j*,*k*,*ℓ*. This odds ratio is the same between the final bistochastic matrix and the original raw adjacency matrix. Thus the IPFP method focuses on relative inter-disease flows and discards the absolute link weights.

### The GloSS filter

We discussed above that the disparity filter had a local approach; focusing on the distribution of link strengths of individual nodes among their immediate in-neighbors or out-neighbors. That is, the disparity filter assesses how likely a link strength is to be a nonrandom fluctuation in the links of an individual node. An alternative approach would be to allow the weights to be distributed globally, while still retaining the degrees fixed. This leads to the Global Statistical Significance (GloSS) filter (Radicchi et al. 2011). The GloSS filter assesses how likely a particular link weight is to be a nonrandom fluctuation in the whole network. This method works as follows. We first fix the network topology, that is, the node degrees and directions. This unweighted network is the substrate for the null model. Denote the empirically-observed weight distribution of links by $\hat {p}(w)$. The null model is constructed by assigning to each link of the substrate network a value randomly drawn from the global empirical distribution $\hat {p}(w)$.

We introduce the auxiliary probability distribution *F*(*s*,*k*), which is the probability that randomly drawing *k* values from the weight distribution $\hat {p}(w)$ will yield values that sum up to *s*. It is straightforward to show that *F*(*s*,*k*) is obtained by convolving $\hat {p}(w)$ with itself *k* times. More simply, we can take the inverse Fourier transform of the *k*-th power of the Fourier transform of the original distribution:

15 $$\begin{array}{*{20}l}{ F(s,k)= \frac{1}{2\pi} \int_{0}^{\infty} \left[\int_{0}^{\infty} \hat{p}(w) e^{i w \phi} dw \right]^{k} e^{-i s \phi} d\phi }\end{array} $$

Now we can apply Bayes’ rule to obtain:

16 $$\begin{array}{*{20}l} P\left(w_{ij}|s_{i}^{\text{out}},k_{i}^{\text{out}},s_{j}^{\text{in}},k_{j}^{\text{in}}\right) &= \hat{p}(w_{ij}) \frac{P\left(s_{i}^{\text{out}},s_{j}^{\text{in}}|w_{ij},k_{i}^{\text{out}},k_{j}^{\text{in}}\right)}{P\left(s_{i}^{\text{out}},s_{j}^{\text{in}}|k_{i}^{\text{out}},k_{j}^{\text{in}}\right)}  \\ &= \hat{p}(w_{ij}) \frac{F\left(s_{i}^{\text{out}}-w_{ij},k_{i}^{\text{out}}-1\right) F\left(s_{j}^{\text{in}}-w_{ij},k_{j}^{\text{in}}-1\right)}{P\left(s_{i}^{\text{out}},s_{j}^{\text{in}}|k_{i}^{\text{out}},k_{j}^{\text{in}}\right)} \end{array} $$

We can thus compute *α*_*ij*_, which is the probability that, under the null, the value of *w*_*ij*_ is greater than a given value. So we arrive at the *p*-value for the observed weight *w*_*ij*_:

17 $$\begin{array}{*{20}l}{ \alpha_{ij}= \frac{\int_{w_{ij}}^{\infty} \hat{p}(w) F\left(s_{i}^{\text{out}}-w,k_{i}^{\text{out}}-1\right) F\left(s_{j}^{\text{in}}-w,k_{j}^{\text{in}}-1\right)dw} {\int_{0}^{\infty} \hat{p}(w) F\left(s_{i}^{\text{out}}-w,k_{i}^{\text{out}}-1\right) F\left(s_{j}^{\text{in}}-w,k_{j}^{\text{in}}-1\right)dw} }\end{array} $$

The method then proceeds by retaining those links whose *p*-values are smaller than a threshold value which sets the significance level. In this paper, we set *α*=0.05.

It is of note that the *p*-values of the links are not necessarily highly correlated with link weights. This feature enables a trade-off between topology and weight. That is, the method has the advantage that it can capture informative links whose weights might not be outstandingly large.

### Link salience

An alternative approach for assessing the importance of a link is link salience (Grady et al. 2012). The link salience method takes a more global approach as compared to the previous methods. The underlying rationale of this method can be intuitively described with an analogy to road networks: if the network represents the network of roads between locations, then the link salience method is trying to partition the links into superhighways and roads (Wu et al. 2006). This method involves viewing the network from the point of view of every single node, and measuring how important a given link is viewed by all nodes. The algorithm works as follows. For a path {*v*_1_,…,*v*_*ℓ*_} between source node *v*_1_ and target node *v*_*ℓ*_, we define the total effective distance as ${\sum \nolimits }_{i=1}^{\ell } 1/w_{v_{i}v_{i+1}}$. So between any pair of nodes, we can define a shortest path as the path that minimizes the effective distances. For each node *v*, we find the shortest paths to every other node. This can be done by standard methods, such as Dijkstra’s algorithm. The shortest-path tree rooted at node *v* can be represented by a binary matrix *T*(*v*) with the same size as the original network. So *T*(*v*)_*ij*_ is 1 if the link from node *i* to node *j* exists on at least one shortest path from *v* to some other node, and *T*(*v*)_*ij*_ is zero otherwise. Finally, the salience of the network is defined as the following matrix: $S=(1/N) {\sum \nolimits }_{v} T(v)$. This matrix gives the link salience values to be used for extracting the network skeleton.

There are several advantageous of this method. First, if the salience of a link is high (that is, close to unity), this means that it participates in most of the shortest-path trees. Thus, viewed from the point of view of the majority of the nodes, this link is important for reaching other nodes. This helps one capture pathways that are critical in reaching certain diseases. For instance, a certain link might not be particularly heavy, yet this method might pick it up because it is essential in reaching a certain disease which is otherwise isolated. Second, this method is highly robust regarding the choice of significance threshold. The distribution of link salience values calculated with the above procedure is bimodal: most links have salience concentrated near zero, a small minority have salience concentrated around unity, and the rest of the links take intermediate salience values. Such a bimodal nature of this distribution considerably facilitates the choice of threshold, because the links that fall in the intermediary region between the two peaks are a negligibly-small minority and any threshold value in this region will retain almost the same set of links. Third, this method enables characterizing the risks associated with *links*, rather than the nodes. The global nature of this approach enables extraction of ‘disease highways’, which are the main multi-disease progression paths. In this paper, we used the threshold value of 0.9 for the salience values. That is, we retain all the links with salience greater than 0.9.

## Comparing networks

In this section we first provide a broad overview of the structure of the constructed networks. We then introduce different network measures for characterizing disease importance and apply these measures to the constructed networks. The measures that we use are in-strength, out-strength, eigenvector centrality, PageRank, Hubs and Authority (the HITS algorithm (Kleinberg 1999)), and betweenness centrality.

### Overview of the function of different networks

Table 3 presents the summary statistics of the networks built with the above methods. For each network, we calculated the number of links, the percentage of links from the raw network that the method retains, and the number of nodes for each network. To describe the connectivity of nodes, we calculate the mean and standard deviation of the out-strengths and out-degrees, which are mathematically identical to those for the in-strengths and in-degrees, respectively. To characterize the relation between the degrees of nodes and their neighbors, we calculate the nearest-neighbor degree statistics as follows. For each node, we find the average out-degree of its out-neighbors. Then we calculate the mean and standard deviation of these values across all nodes. This measure quantifies the nearest-neighbor degree correlations of the network. For each network, we denote the average prevalence of the diseases kept by the method by 〈*P*〉. For each disease, we can find the average prevalence of its neighboring diseases. The average over all these average values is denoted by $P_{nn}^{\text {out}}$ if we use the out-neighbors for calculations, and $P_{nn}^{\text {in}}$ if we use the in-neighbors. Finally, we report a measure of homophily, calculated as the correlation between the prevalence of diseases and the average prevalence of the neighbors of diseases. This assortativity coefficient can be calculated using either the out-neighbors or the in-neighbors. The resulting coefficients are denoted by $r_{P}^{\text {out}}$ and $r_{P}^{\text {in}}$, respectively. These measures can also be visibly investigated from Figs. 5 and 6. Every constructed network is a subsample of the raw network topologically (disregarding the link weights). But there is no particular relation between the constructed networks, that is, one cannot be derived from the other, because the methods employ different filtering rationales. The OER, *ϕ*, and IPFP methods, assign new weights to the inter-disease links, which are different from the raw link weights which simply denote the number of diagnosis successions. In contrast, in the DF, Gloss, and Salience methods, filtering techniques are applied only to decide which links of the raw network to retain and which to discard, and the weights of the retained links remain intact.

**Table 3:** Summary statistics of the constructed networks

	Raw	OER	phi	DF	IPFP	GloSS	Salience
# links	434418	96837	113715	36043	20745	9587	678
% link overlap with the raw network	100	22.3	26.2	8.30	4.87	2.21	0.16
# of nodes retained	912	837	911	832	870	280	711
〈*k*^out^〉=〈*k*^in^〉	460	115	124	43.3	23.8	34.2	0.954
std (*k*^out^)=std(*k*^in^)	229	107	105	24.8	14.5	46.5	0.211
$\langle k_{nn}^{\text {out}} \rangle $	603	227	194	80.1	29.11	127	0.42
std$(k_{nn}^{\text {out}})$	85.5	53.2	38.8	7.52	8.52	49.8	0.49
〈*P*〉	7160	7801	7169	7848	5751	16837	6478
median (*P*)	1439	1771	1445	1812	1384	9531	1341
$\langle P_{nn}^{\text {out}} \rangle $	14554	24100	15443	55724	2957	63539	122077
$\langle P_{nn}^{\text {in}} \rangle $	15258	28665	16761	11059	2845	63820	2046
$\langle r_{P}^{\text {out}} \rangle $	-0.295	-0.197	-0.027	-0.231	0.227	-0.482	0.038
$\langle r_{P}^{\text {in}} \rangle $	-0.259	-0.240	-0.071	0.102	0.164	-0.421	0.227

The 〈·〉 operator denotes average, and the *nn* subscript denotes nearest-neighbor

**Fig. 4:**
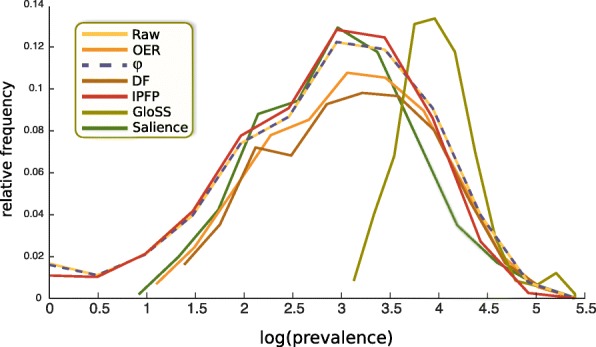
Visualizing Prevalences. The distribution of the ln(prevalence) of the diseases in different constructed networks sheds light on their selection criteria. The GloSS method retains disproportionately high-prevalence diseases, the IPFP method discards disproportionately high-prevalence diseases, and the OER, DF, and salience methods, discard disproportionately low-prevalence diseases

**Fig. 5:**
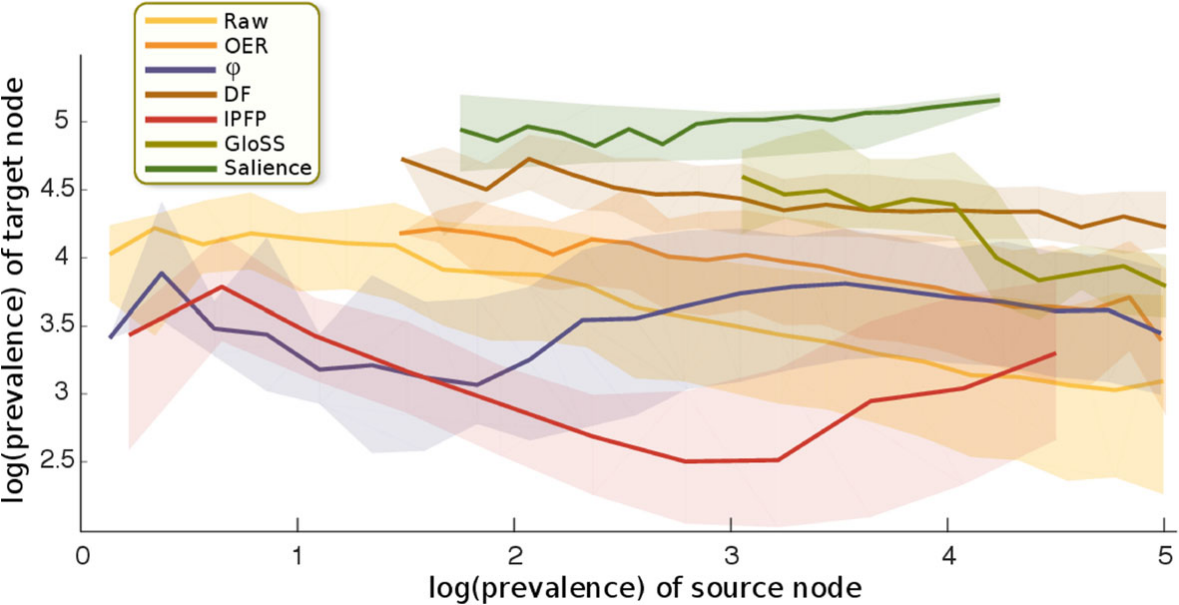
Comparing prevalences of neighboring nodes. The horizontal axis pertains to the prevalence of the diseases, and the vertical axis depicts the distribution of the prevalences of their out-neighbors. The thick lines denote median values and the shades demarcate 0.25 and 0.75 quantiles. The axes are natural-logarithmic

**Fig. 6:**
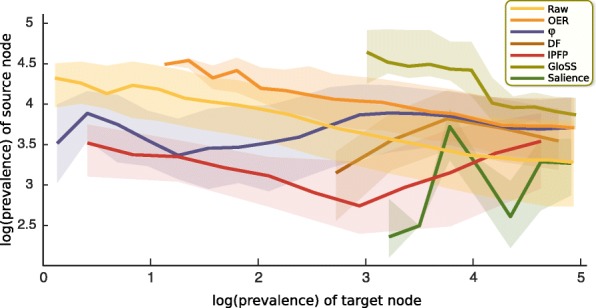
Comparing prevalences of neighboring nodes. The horizontal axis pertains to the prevalence of the diseases, and the vertical axis depicts the distribution of the prevalences of their in-neighbors. The thick lines denote median values and the shades demarcate 0.25 and 0.75 quantiles. The axes are natural-logarithmic

From Table 3 we can obtain quick insight into the differences between the function of different methods. For the raw network, the correlations between the prevalence of diseases with their in-neighbors and that with their out-neighbors, denoted by $r_{P}^{\text {in}}$ and $r_{P}^{\text {out}}$ respectively, are *both* negative. This is also evident from the negative slope of the curves in Figs. 5 and 6 corresponding to the raw network. Also, the prevalence distribution of the raw network, presented in Fig. 4, shows that the distribution of the prevalences has a lognormal-like shape, so most diseases have small prevalence, and high-prevalence diseases are relatively less common. Combining these two observations, we deduce that the connections are strongly disassortative and mutual. That is, the network has a core-periphery structure in which a few highly-prevalent nodes preferentially connect to many low-prevalence nodes, and vice versa.

The OER and *ϕ* methods retain comparable portions of the links, but the OER network discards about 10% of the nodes. We manually verified that all the diseases that the OER method discards are in-fact extremely low-prevalence. This is also visible in Fig. 4, where the left tail of the OER curve begins around 12, falling in the 6th percentile of the prevalence distribution of the raw network. So we observe that the OER network discards *all* the diseases in the bottom 5 percentile of prevalence. From Figs. 5 and 6, we also observe that the low-prevalence diseases are discarded by the OER method. We also observed that the curves corresponding to the OER method have negative slopes, similar to the raw network. This indicates that the OER method retains the structural core-periphery property discussed above. For the *ϕ* network, correlation between the prevalence of diseases with their in-neighbors and that with their out-neighbors are close to zero. This means that the disease pairs in the *ϕ* network neither exhibit homophily nor heterophily in prevalence. This is visible in Figs. 5 and 6, where the curves pertaining to the *ϕ* method have overall slope close to zero.

The DF network is considerably sparser than OER though it retains roughly the same number of nodes. In contrast to the OER and *ϕ* networks, the DF network exhibits a high imbalance between $\langle P_{nn}^{\text {out}} \rangle $ and $\langle P_{nn}^{\text {in}} \rangle $. This means that on average, the average prevalence of out-neighbors of nodes is five times greater than the average prevalence of the in-neighbors of nodes. This is expected by construction, because the way the DF network was built was to retain disproportionately-large out-links of each node, and discard the out-links with smaller weights. So for a typical disease, heavier out-links are systematically selected, and these are the links that typically point to high-prevalence diseases. In other words, high-prevalence diseases are the ones which appear with high frequency among either the in-neighbors or the out-neighbors of a typical disease, and the DF method systematically discards light out-links without doing the same to the in-links, thereby creating an imbalance. Consequently, high-prevalence nodes become more likely to appear among the out-neighbors as before. This asymmetry is also evident from the mismatch between the signs of $r_{P}^{\text {in}}$ and $r_{P}^{\text {out}}$. Moreover, as it is visible in Fig. 4, the DF method discards diseases with low prevalence almost entirely, which is a feature it shares with the OER method.

The IPFP network is sparser than OER, *ϕ*, and DF networks. The average prevalence of the diseases that IPFP retains is lower than the other three methods. Same is true for the median of the prevalences. This means that the IPFP filter discards disease with extremely high prevalence. This is also evident from the tail behavior of the distribution of log(prevalence) in Fig. 4, where the tail of the IPFP curve visibly plummets below the other curves. The $\langle P_{nn}^{\text {out}} \rangle $ and $\langle P_{nn}^{\text {in}} \rangle $ values of the IPFP network are an order of magnitude smaller than the corresponding values in the OER, *ϕ*, and DF networks. Thus the core-periphery feature is nonexistent in the IPFP network, and the prevalences of the disease pairs are on average less unequal than other networks. Moreover, the IPFP method rewards intermediacy. As Fig. 4 shows, the intermediate-prevalence diseases constitute a higher fraction of the IPFP method as compared to the raw network.

The GloSS filter has a unique property: the average prevalence is twice as high as the raw network. So the GloSS method has retained disproportionately high-prevalence diseases. This is also evident from Fig. 4. The prevalence curve pertaining to the Gloss method is shifted to the far right. In fact, the lowest prevalence in the GloSS network is 1176, which in the raw network, is at the 47 percentile. So the Gloss network discards about half of the diseases, and it suffers from this problem much more severely than OER and DF methods. The values of $\langle P_{nn}^{\text {out}} \rangle $ and $\langle P_{nn}^{\text {in}} \rangle $ are also much greater than the average and median values of *P*. Moreover, $r_{P}^{\text {in}}$ and $r_{P}^{\text {out}}$ are strongly negative. These indicate a strong mutual core-periphery structure. This feature was also present in the raw network as discussed above, but is markedly accentuated by the GloSS method. The GloSS method discards low-prevalence diseases, and retains medium and high-prevalence diseases. As Figs. 5 and 6 illustrate, in the remaining network, the out-links of high-prevalence diseases are preferentially towards medium-prevalence diseases, and conversely, the out-links of medium-prevalence diseases are preferentially towards high-prevalence diseases.

The Salience network is the most sparse, retaining less than 2.5% of all the links in the raw data. The Salience network is small by construction because it seeks to retain a small fraction of links that would capture the macro skeleton of the network. Since according to Table 3 the number of links is smaller than the number of nodes in this network, we deduce that the Salience network is disconnected. Via visualization we verified that the Salience network is in fact segregated to disjoint connected components. The Salience method, like the OER, DF, and GloSS methods, is biased towards high-prevalence diseases. However, the degree to which the Salience method suffers from this bias is comparable to the OER and DF methods, and is not as severe as the Gloss method. Another notable uniqueness of the Salience method is the great difference between $\langle P_{nn}^{\text {out}} \rangle $ and $\langle P_{nn}^{\text {in}} \rangle $, with the former being about 60 times greater than the latter. This was the case for the DF network too, but this ratio was about five, which is a much smaller difference than in the Salience network. To investigate if the high skew of the prevalence distribution is disproportionately affecting the averages, we repeated the calculations using the median instead of average, and we observed the same pattern both for DF and salience networks. This indicates a highly-unequal three-level structure in the Salience network: the Salience network comprises many locally-core-periphery substructures, where the peripheral nodes preferentially connect to the core nodes, but the core nodes do not reciprocate. Some of the core nodes connect to other core nodes, and some of them do not. That is, there are three types of nodes in the Salience network: (i) core nodes with high in-strength (received by many unilateral links from small peripheral nodes), (ii) nodes with high in-strength and intermediate out-strength (the in-flow comes form many peripheral nodes, the out-flow goes into the core nodes from the first category), and (iii) the small peripheral nodes who do not receive any in-flow and form unilateral links towards the former two categories. This topology can be simply conceptualized as follows: consider a star graph with many leafs, all unilaterally linking to the central node. Now suppose we take a fraction of the leafs, and for each of them introduce some new leaf nodes that unilaterally connect to them, turning them into mini-authorities. This structure exhibits the correlation properties observed in Table 3 for the Salience network. We also visually verified this hypothesis. Note that the lack of reciprocation from high-prevalence nodes is in contrast to the raw and OER networks, which although highly unequal, comprised mostly-bidirectional links.

### Applicability of different methods

To summarize, the OER and *ϕ* networks focus on disease-disease relationships in the usual statistical way, that is, in the absence of network structure. If the task of a study is to investigate the comorbidity between a certain pair of diseases, then these methods are suitable. The OER network disproportionately discards diseases with low prevalences, and the *ϕ* network disproportionately discards disease pairs with highly-unequal prevalences. The DF network is better if the questions of comorbidity are being formulated conditional on having developed certain diseases first, and if one wants to compare between the risks of different diseases that succeed the given initial diseases. The DF method has the disadvantage that it discards diseases with low prevalence. The IPFP method has an egalitarian approach which controls for disease prevalence. This method prevents the results from being dominated by high-prevalence diseases. It asks if all the diseases had the same in-flow and the same out-flux, what would be the best estimate of the comorbidity matrix, given the information on the empirical matrix? In other words, the IPFP method investigates comorbidity patterns controlled for individual disease prevalences. The Gloss method assesses link weight fluctuations on a global level. So this method is preferred when the task of the study is to compare comorbidity links globally, not focusing on a particular disease. That is, if the emphasis of the study is on links rather than the nodes. The Salience method is only relevant for disease trajectories globally and is not suitable for studying comorbidity statistics. The Salience method is suitable if, for instance, one would like to investigate the expected distance between certain diseases, that is, how many intermediate diseases it would typically take to develop disease B having developed disease A. The OER, *ϕ*, and DF methods have a local approach. The Gloss filter and the Salience method have global approaches. The IPFP method has a meso-scale approach.

## Example applications

In what follows we calculate several conventional measures of node importance. We investigate the agreement between different networks on the importance profile of diseases. We denote the diseases by 3-digit ICD9 codes.

### Different measures for node importance

**Node Strength.** For directed weighted networks, we can use the out-strength and in-strength to characterize nodal connectivity, as discussed above. Table 4 presents the top 5 diseases with highest in-strength, and Table 5 presents the top 5 diseases with highest out-strength.

**Table 4:** Top 5 diseases with highest in-strength in different networks

Raw	OER	*ϕ*	DF	IPFP	GloSS	Salience
285 anemia (unspecified)	474 Chronic disease of tonsils and adenoids	285 anemia (unspecified)	285 anemia (unspecified)	766 long gestation	285 anemia (unspecified)	366 cataract
041 bacterial infection (unspecified)	648 other conditions complicating pregnancy, childbirth or puerperium	366 cataract	041 bacterial infection (unspecified)	773 Hemolytic disease of fetus or newborn due to isoimmunization	041 bacterial infection (unspecified)	664 trauma to perineum and vulva during delivery
276 fluid electrolyte disorders	664 trauma to perineum and vulva during delivery	584 acute kidney failure	276 fluid electrolyte disorders	763 fetus or newborn affected by other complications of labor and delivery	276 fluid electrolyte disorders	401 essential hypertension
366 cataract	654 pelvis abnormalities (e.g., previous cesarean)	272 lipoid metabolism disorders	366 cataract	764 slow fetal growth and fetal malnutrition	427 Cardiac dysrhythmias	272 lipoid metabolism disorders
584 acute kidney failure	381 otitis media and eustachian tube disorders	041 bacterial infection (unspecified)	584 acute kidney failure	772 fetal and neonatal hemorrhage	414 chronic ischemic heart disease	414 chronic ischemic heart disease

**Table 5:** Top 5 diseases with highest out-strength in different networks

Raw	OER	*ϕ*	DF	IPFP	GloSS	Salience
401 essential hypertension	664 trauma to perineum and vulva during delivery	401 essential hypertension	401 essential hypertension	887 traumatic amputation of arm	401 essential hypertension	401 essential hypertension
414 chronic ischemic heart disease	474 Chronic disease of tonsils and adenoids	366 cataract	366 cataract	673 Obstetrical pulmonary embolism	414 chronic ischemic heart disease	244 acquired hypothyroidism
272 lipoid metabolism disorders	663 umbilical cord complications during delivery	414 chronic ischemic heart disease	414 chronic ischemic heart disease	896 traumatic amputation of foot	272 lipoid metabolism disorders	496 chronic airway obstruction (unclassified)
366 cataract	658 problems of amniotic cavity membranes	272 lipoid metabolism disorders	272 lipoid metabolism disorders	817 multiple fractures of arm bone	250 Diabetes mellitus	648 other conditions complicating pregnancy, childbirth or puerperium
250 Diabetes mellitus	656 other fetal and placental problems	250 Diabetes mellitus	250 Diabetes mellitus	897 traumatic amputation of leg	427 Cardiac dysrhythmias	413 Angina pectoris

**Eigenvector centrality.** A basic measure to characterize the centrality of nodes in a network is the eigenvector centrality (Bonacich 1987). The basic intuition behind this measure is that important nodes are those that are connected to other important nodes. This yields a self-consistent linear set of equations that yields the centrality scores of nodes. Table 6 presents the top 5 diseases with highest eigenvector centrality for different networks.

**Table 6:** Top 5 diseases with highest eigenvector centrality in different networks

Raw	OER	*ϕ*	DF	IPFP	GloSS	Salience
041 bacterial infection (unspecified)	664 trauma to perineum and vulva during delivery	654 trauma to perineum and vulva during delivery	285 anemia (unspecified)	651 multiple gestation	041 bacterial infection (unspecified)	401 essential hypertension
285 anemia (unspecified)	412 old myocardial infarction	412 old myocardial infarction	041 bacterial infection (unspecified)	647 other infections complicating pregnancy	285 anemia (unspecified)	366 cataract
276 fluid electrolyte disorders	585 chronic kidney disease	585 chronic kidney disease	780 general symptoms	652 malposition and malpresentation of fetus	427 Cardiac dysrhythmias	664 trauma to perineum and vulva during delivery
780 general symptoms	648 other conditions complicating pregnancy, childbirth or puerperium	648 other conditions complicating pregnancy, childbirth or puerperium	276 fluid electrolyte disorders	641 Antepartum hemorrhage	276 fluid electrolyte disorders	272 lipoid metabolism disorders
427 Cardiac dysrhythmias	654 pelvis abnormalities (e.g., previous cesarean)	654 pelvis abnormalities (e.g., previous cesarean)	599 urinary tract infection	663 umbilical cord complications during delivery	414 chronic ischemic heart disease	285 anemia (unspecified)

The rankings of top nodes show the differences between the networks constructed by the different methods. It is of note that eigenvector centrality tends to capture nodes with high *in*-strength. The OER and IPFP methods predominantly pick up pregnancy-related diagnoses, whose comorbidity links (preceding or following other diseases or conditions) to several different categories of diseases are well-researched (Desai et al. 2007; James et al. 2005; Brabin et al. 2001; Kittner et al. 1996). The major difference between pregnancy-related ICD codes (630-679) and other categories is cohesion. As shall be discussed below in Tables 19 and 20, the aggregated in-strength and out-strength of this category of nodes is not high as compared to other categories (it ranks among the bottom 5 in both cases), but interestingly, in terms of the weights of within-category links, this category has an outstandingly large share (it ranks second, after the diseases of the circulatory system). This means that the pregnancy-related nodes form a cohesive subnetwork. This dense clique-like structure is comprised of disease with intermediate-level prevalence with prevalence values all relatively close to one another. The OER coefficient is high for these disease pairs because in addition to high co-occurrence, all diseases have intermediate levels of prevalence, so the overestimation and underestimation tendencies of the OER method are not encountered. Another family of diseases that frequently have high values of eigenvector centrality in all network construction methods is the family of 28X diseases, which are the diseases of the blood and blood-forming organs. Notable diseases in this family include different types of anemia, Haemophilia, and diseases of white blood cells. Iron deficiency anemias (280) and ‘Other and unspecified anemias’ (285) appear consistently higher than Haemophilia. This is mainly caused by the high prevalence of 280 and 285 anemias (with prevalences 40,000 and 107,000, respectively), as compared to Haemophilia, whose prevalence is about 10,000 in our data set.

Table 7 presents the mutual correlation coefficients for the eigenvector centrality of nodes computed for different networks. The IPFP method seems uncorrelated or negatively correlated with the other methods. Most methods are strongly correlated in terms of eigenvector centrality. The Salience method focuses on trajectories and distorts the degrees, so the eigenvector centrality of this method is weakly correlated with that of the other methods. The IPFP method changes the strengths and assigns new link weights after controlling for disease prevalences. The IPFP method has a strong negative association with the GloSS method, because by construction, the GloSS method rewards high-prevalence diseases and the IPFP method does the converse.

**Table 7:** The correlation matrix for the eigenvector centrality of nodes between different networks

	Raw	OER	*ϕ*	DF	IPFP	GloSS	Salience
Raw	1	0.799	0.783	0.424	-0.205	0.505	0.120
OER	0.799	1	0.996	0.558	0.017	0.330	0.212
phi	0.783	0.996	1	0.554	0.093	0.315	0.214
DF	0.424	0.558	0.554	1	-0.223	0.605	0.372
IPFP	-0.205	0.017	0.093	-0.223	1	-0.414	-0.003
GloSS	0.505	0.330	0.315	0.605	-0.414	1	0.275
Salience	0.120	0.212	0.214	0.372	-0.003	0.275	1

**PageRank.** The second centrality measure that we use is the PageRank (Brin and Page 1998). The PageRank algorithm was originally used to characterize the importance of websites. This algorithm basically quantifies the likelihood that a person clicking randomly on links will arrive at a given website (Xing and Ghorbani 2004). This method simulates random walks on the network, with a damping factor that characterizes the probability that the walk terminates at any step and restarts at a node chosen uniformly at random. We set the damping factor equal to 0.85, which is conventional in the literature. Table 8 presents the results for the top 5 diseases with highest PageRank for different networks. An intuitive approximation is that PageRank tends to focus on nodes with high *in*-strength (Fortunato et al. 2006). This is confirmed by comparing Table 8 with Table 4; many of the top nodes are common between these two tables. Comparing Table 8 with Table 6, we observe that IPFP is returning different results—mostly conditions pertaining to the perinatal period. We point out that most of these diseases are not particularly highly-connected nodes in the raw network. Motivated by this observation, we can gain intuition about how IPFP works by noting that, when normalization of rows or columns is performed at each stage, nodes with large degrees can lose their weight if their neighbors are also of large degree. For example, consider the row-normalization step, where rows (and similarly columns) are normalized to add up to unity. At this step, a row that corresponds to a node with out-degree 100 who is connected with heavy and equal out-links to its 100 out-neighbors will become identical to a row corresponding to a node with out-degree 3 who is connected with light and equal out-links to its 3 out-neighbors. In other words, this method is capturing something that is inherently of a different nature than every other method considered here. Table 9 presents the correlation values for PageRank scores in different networks. Similar to the case of eigenvector centrality, IPFP is negatively correlated with every other method. This observation highlights the difference between IPFP and other methods, and invites more investigation into what this method extracts.

**Table 8:** Top 5 diseases with highest PageRank in different networks

Raw	OER	*ϕ*	DF	IPFP	GloSS	Salience
285 anemia (unspecified)	585 chronic kidney disease	285 anemia (unspecified)	285 anemia (unspecified)	773 Hemolytic disease of fetus or newborn due to isoimmunization	285 anemia (unspecified)	366 cataract
041 bacterial infection (unspecified)	664 trauma to perineum and vulva during delivery	366 cataract	041 bacterial infection (unspecified)	766 long gestation	041 bacterial infection (unspecified)	401 essential hypertension
276 fluid electrolyte disorders	428 heart failure	272 lipoid metabolism disorders	276 fluid electrolyte disorders	762 complications of placenta affecting newborn	276 fluid electrolyte disorders	664 trauma to perineum and vulva during delivery
584 acute kidney failure	648 other conditions complicating pregnancy, childbirth, or puerperium	584 acute kidney failure	584 acute kidney failure	769 Respiratory distress syndrome in newborn	427 Cardiac dysrhythmias	272 lipoid metabolism disorders
780 general symptoms	474 Chronic disease of tonsils and adenoids	041 bacterial infection (unspecified)	366 cataract	652 malposition and malpresentation of fetus	414 chronic ischemic heart disease	285 anemia (unspecified)

**Table 9:** The correlation matrix for the PageRank of nodes between different networks

	Raw	OER	*ϕ*	DF	IPFP	GloSS	Salience
Raw	1	0.371	0.986	0.966	-0.528	0.892	0.377
OER	0.371	1	0.408	0.250	-0.525	0.039	0.129
phi	0.986	0.408	1	0.961	-0.491	0.857	0.430
DF	0.966	0.250	0.961	1	-0.369	0.905	0.386
IPFP	-0.528	-0.525	-0.491	-0.369	1	-0.217	-0.084
GloSS	0.892	0.039	0.857	0.905	-0.217	1	0.228
Salience	0.377	0.129	0.430	0.386	-0.084	0.228	1

Table 9 is also informative regarding the function of different methods. The PageRank scores of the raw network have a strong positive correlation with those in the *ϕ*, DF, and GloSS networks. The IPFP method shows a negative correlation, similar to the case of eigenvector centrality, because it punishes diseases with high prevalence and assigns a low strength to them, as discussed before. The Salience method constructs a network comprising entirely of non-mutual links and distorts the in-strength and out-strength patterns. The in-strength of most nodes are mapped to zero, because, as we discused above, the Salience method comprises mostly of highly-unequal substructures with peripheral nodes unidirectionally connected to core nodes. As mentioned above, the PageRank scores of nodes can be approximated with their in-strength (Fortunato et al. 2006). Since the Salience method maps the in-degrees of many nodes to zero (retaining only their out-links to highly-prevalent core nodes, as discussed above), the concomitant distortion in in-strengths results in a lower correlation between the PageRank of the Salience method and other networks.

**Hubs and authority.** An alternative way we could characterize the importance of nodes in terms of in-flow and out-flow is to employ the HITS algorithm (Kleinberg 1999). The HITS algorithm is a simple and intuitive method that was originally devised to characterize the rankings of websites. This algorithm focuses on simultaneously finding *hubs* and *authorities* on the web. In that context, a hub is a website that is influential in directing users towards other highly-ranked websites, and an authority is a website which gets directed to by highly-ranked nodes. In the context of diseases, a hub would be a disease which, if developed, increases the risks of developing other diseases. An authority would be a disease that follows many other diseases. Table 10 presents the results for the top 5 diseases in terms of hubness in different networks. Comparing the results in Table 10 with those of Table 6, we observe that the hub-ness scores for all categories correlate highly with those of eigenvector centrality, except DF and Salience. Table 11 presents the correlation matrix for hubness. The hubness score is important in comorbidity studies because the hubs that the HITS algorithm nominates are universal senders, and in the context of comorbidity studies, these would pertain to diseases that substantially increase the risk of many other diseases, demanding more prevention and care. Table 11 shows that there is good agreement between the hub scores of different methods, so despite their structural differences, the hub score is robust and can be reliably used. The two usual exceptions are present here as well: the IPFP method, and the Salience method. These are expected because their tasks are different: the hubness scores of the IPFP method pertain to an alternative inflow-outflow comorbidity matrix where the prevalences are controlled for, and the Salience method only focuses on distances and trajectories rather than actual disease-disease relations. Table 12 presents the top 5 nodes for authority, and Table 13 presents the correlation matrix for authority scores. For the authority index too, there is good agreement between every method except IPFP and Salience.

**Table 10:** Top 5 diseases with highest hub-ness scores in different networks, according to the HITS algorithm

Raw	OER	*ϕ*	DF	IPFP	GloSS	Salience
041 bacterial infection (unspecified)	664 trauma to perineum and vulva during delivery	664 trauma to perineum and vulva during delivery	276 fluid electrolyte disorders	666 postpartum hemorrhage	041 bacterial infection (unspecified)	225 Benign neoplasm of brain and other parts of nervous system
285 anemia (unspecified)	366 cataract	366 cataract	780 general symptoms	656 other fetal and placental problems	427 Cardiac dysrhythmias	303 alcohol dependence syndrome
780 general symptoms	648 other conditions complicating pregnancy, childbirth, or puerperium	648 other conditions complicating pregnancy, childbirth, or puerperium	535 Gastritis and duodenitis	658 problems of amniotic cavity membranes	285 anemia (unspecified)	344 other paralytic syndromes
276 fluid electrolyte disorders	656 other fetal and placental problems	656 other fetal and placental problems	285 anemia (unspecified)	628 female infertility	250 Diabetes mellitus	270 Disorders of amino-acid transport metabolism
250 Diabetes mellitus	250 Diabetes mellitus	250 Diabetes mellitus	486 Pneumonia	634 spontaneous abortion	272 lipoid metabolism disorders	324 Intracranial and intraspinal abscess

**Table 11:** The correlation matrix for the hubness score of nodes between different networks, according to the HITS algorithm

	Raw	OER	*ϕ*	DF	IPFP	GloSS	Salience
Raw	1	0.763	0.741	0.915	-0.054	0.408	0.132
OER	0.763	1	0.995	0.560	0.243	0.375	-0.199
*ϕ*	0.741	0.995	1	0.510	0.296	0.366	-0.222
DF	0.915	0.560	0.510	1	-0.404	0.349	0.212
IPFP	-0.054	0.243	0.296	-0.404	1	-0.180	-0.217
GloSS	0.408	0.375	0.366	0.349	-0.180	1	-0.142
Salience	0.132	-0.199	-0.222	0.212	-0.217	-0.142	1.000

**Table 12:** Top 5 diseases with highest authority scores in different networks, according to the HITS algorithm

Raw	OER	*ϕ*	DF	IPFP	GloSS	Salience
285 anemia (unspecified)	585 chronic kidney disease	585 chronic kidney disease	285 anemia (unspecified)	652 malposition and malpresentation of fetus	285 anemia (unspecified)	401 essential hypertension
041 bacterial infection (unspecified)	664 trauma to perineum and vulva during delivery	664 trauma to perineum and vulva during delivery	041 bacterial infection (unspecified)	656 other fetal and placental problems	041 bacterial infection (unspecified)	765 Disorders relating to short gestation and low birthweight
276 fluid electrolyte disorders	411 old myocardial infarction	411 old myocardial infarction	276 fluid electrolyte disorders	651 multiple gestation	427 Cardiac dysrhythmias	041 bacterial infection (unspecified)
780 general symptoms	428 heart failure	428 heart failure	780 general symptoms	647 other infections complicating pregnancy	276 fluid electrolyte disorders	762 complications of placenta affecting newborn
427 Cardiac dysrhythmias	648 other conditions complicating pregnancy, childbirth, or puerperium	648 other conditions complicating pregnancy, childbirth, or puerperium	599 urinary tract infection	648 other conditions complicating pregnancy, childbirth, or puerperium	272 lipoid metabolism disorders	769 Respiratory distress syndrome in newborn

**Table 13:** The correlation matrix for the authority score of nodes between different networks, according to the HITS algorithm

	Raw	OER	*ϕ*	DF	IPFP	GloSS	Salience
Raw	1	0.806	0.786	0.410	-0.113	0.645	0.051
OER	0.806	1	0.994	0.556	0.146	0.355	0.084
phi	0.786	0.994	1	0.553	0.199	0.325	0.084
DF	0.410	0.556	0.553	1	-0.107	0.587	0.150
IPFP	-0.113	0.146	0.199	-0.107	1	-0.461	-0.015
GloSS	0.645	0.355	0.325	0.587	-0.461	1	0.139
Salience	0.051	0.084	0.084	0.150	-0.015	0.139	1

**Betweenness centrality.** Distance-based network measures capture different aspects of how essential a node is in the reachability between other pairs of diseases. Here we use betweenness centrality which characterizes the number of shortest paths between different disease pairs that pass through each given disease. There might be a disease that separates a dense module of diseases from the whole network, such that one would develop the diseases within the module only when one first develops this gate-keeper disease. Or, conversely, after developing a disease within the module, subsequent diseases outside the module occur typically after this gate-keeper disease is developed. Such a disease would have a high betweenness centrality. Another type of nodes that are typically characterized by high betweenness centrality are the core nodes in strong core-periphery structures, because to go from one peripheral node to another, one has to pass through the core nodes. So this measure is helpful in detecting this structural feature of diseases. Table 14 presents the top 5 nodes with highest betweenness centrality in the constructed networks. In the raw, OER, *ϕ*, DF, and Gloss networks, the top nodes are those with extremely high prevalence. The above-discussed core-ness underpins the high betweenness centrality of these nodes. The Salience method assigns high betweenness centrality scores to conditions pertaining to the perinatal period, which is potentially related to the first typical case of high betweenness mentioned above. That is, certain prenatal conditions act as gatekeeping conditions between conditions before birth and conditions after birth. The results of the IPFP method are less intuitive, consistent with the results for the previous measures. Table 15 presents the correlations across different networks.

**Table 14:** Top 10 diseases with highest betweenness centrality

Raw	OER	*ϕ*	DF	IPFP	GloSS	Salience
272 lipoid metabolism disorders	366 cataract	366 cataract	654 trauma to perineum and vulva during delivery	032 diphtheria	041 bacterial infection (unspecified)	401 essential hypertension
276 fluid electrolyte disorders	276 nondependent abuse of drugs	664 trauma to perineum and vulva during delivery	789 Other symptoms involving abdomen and pelvis	073 Ornithosis	285 anemia (unspecified)	779 ill-defined conditions originating in prenatal period
250 Diabetes mellitus	276 episodic mood disorders	474 chronic disease of tonsils and adenoids	466 Acute bronchitis and bronchiolitis	827 ill-defined fractures of lower limb	276 fluid electrolyte disorders	771 infections specific to prenatal period
041 bacterial infection (unspecified)	414 chronic ischemic heart disease	414 chronic ischemic heart disease	474 Chronic disease of tonsils and adenoids	004 Shigellosis	427 Cardiac dysrhythmias	770 respiratory conditions of fetus or newborn
285 anemia (unspecified)	272 lipoid metabolism disorders	428 heart failure	041 bacterial infection (unspecified)	254 Diseases of thymus gland	280 iron deficiency anemias	648 other conditions complicating pregnancy, childbirth, or puerperium

**Table 15:** Correlation between the betweenness centrality of nodes across constructed networks

	Raw	OER	phi	DF	IPFP	GloSS	Salience
Raw	1	0.760	0.427	0.494	-0.482	0.638	0.150
OER	0.760	1	0.637	0.530	-0.379	0.217	0.161
phi	0.427	0.637	1	0.457	-0.168	0.237	0.146
DF	0.494	0.530	0.457	1	-0.153	0.289	0.018
IPFP	-0.482	-0.379	-0.168	-0.153	1	-0.187	-0.055
GloSS	0.638	0.217	0.237	0.289	-0.187	1	0.112
Salience	0.150	0.161	0.146	0.018	-0.055	0.112	1

### Example application: the role of disease prevalence

To get a better intuition into the network measures that we used to characterize diseases, we investigate the correlation between each network measure and disease prevalence in every constructed network. The results are presented in Table 16. In the raw network, every measure has a strong positive association with disease prevalence. Thus highly-prevalent diseases such as diabetes and hypertension receive a high score no matter the network measure used to characterize the diseases. Same is true for the DF and GloSS networks. In contrast, for the IPFP network, prevalence is negatively correlated with every network measure except hub and authority, and for the latter two the association is close to zero. For the Salience network, the correlation between the hub index and prevalence is negative. Together with the positivity of the correlation between the authority index and prevalence, this indicates that in the Salience network, high-prevalence nodes disproportionately receive in-flows and do not reciprocate. This is in contrast with every other method, where both of these correlations are positive, meaning that high-prevalence nodes are characterized by both large in-flows and out-flows.

**Table 16:** The correlation between the network measures of diseases and disease prevalence, for different constructed networks

	PageRank	Eig centrality	Authority	Hub	Betweenness centrality	In-strength	Out-strength
Raw	0.794	0.38	0.383	0.395	0.768	0.804	0.946
OER	0.375	0.554	0.56	0.597	0.8	0.233	0.249
phi	0.819	0.557	0.563	0.597	0.708	0.834	0.954
DF	0.776	0.679	0.747	0.388	0.449	0.79	0.947
IPFP	-0.371	-0.067	0.02	0.016	-0.326	-0.367	-0.396
GloSS	0.704	0.641	0.523	0.749	0.678	0.731	0.909
Salience	0.548	0.568	0.257	-0.151	0.265	0.536	0.348

Element *i*,*j* of this table is the correlation between measure *j* and disease prevalence in the constructed network *i*

Note that the exact value of prevalence for each disease cannot be recovered from the networks alone. This is due to the existence of disease progression trajectories with length greater than two. If every patient had a registry of form A →B, that is, only two diseases, then the prevalence of each disease would be simply the sum of the out-strength and in-strength of its corresponding node in the raw network. But because many instances of higher-order records such as A →*B*→C exist, and those with greater lengths, the prevalence information is lost. If we conceptualize the weighted links in the raw network as distinct links with unit weight, then in this picture, each link would represent exactly one patient if every disease trajectory had length one (that is, in the form A →B). But due to the presence of higher-order trajectories, more than one link can together pertain to a single patient, thus the prevalence information lost. However, Table 16 shows a strong correlation between the out-degree of diseases (about 0.95) and their prevalence. So if we did not have the prevalence data, we could use out-degree as a proxy for prevalence. It would be interesting to investigate if this correlation pattern between various network measures and prevalence would be replicated using data from other regions of the globe.

### Example application: shared genes and protein-protein Interactions

We can use the results to investigate the relation to previous studies on disease networks, which in addition to comorbidity observations, incorporate other disease-disease linkages into the analysis. For example, in Ref. Park et al. (2009), the protein–protein interaction (PPI) and coexpression networks and the inter-disease network of shared genes are compared to the comorbidity records from US Medicare claims. Many observed comorbidity patterns are therefore linked to the shared PPIs and the shared genes of the diseases. For example, the significant comorbidity between Alzheimer’s disease and myocardial infarction is linked to their shared ACE and APOE genes. As another example, a PPI between the genes associated with the autonomic nervous system disorder and the carpal tunnel syndrome (IKBKAP and TTR, respectively) is suggested to contribute to the statistically-significant comobrnidity between these two diseases. We can investigate how the results of Ref. Park et al. (2009) are reflected in our constructed networks. Because of the link-focused nature of these results, we expect the local methods to be relevant here, which include the OER measure, the *ϕ* coefficient, and the DF method. In our data set, there are 747 cases where the Alzheimer’s disease is diagnosed following myocardial infarction. All three methods deem this link as significant. There were 506 cases where, conversely, Alzheimer’s disease preceded. Only the DF method deemed this direction of the link as significant, which indicates that myocardial infarction attracts a significant share of the out-strength of the Alzheimer’s disease. This implies that conditional on having developed the Alzheimer’s disease, there is an elevated chance of later developing myocardial infarction. There were 38 cases were autonomic nervous system disorder is diagnosed prior to the carpal tunnel syndrome, and OER and *ϕ* deem this link as significant. There are 47 cases were the carpal tunnel syndrome precedes, and neither of the methods deem this direction of the link as significant. This highlights the strength of the directed characterization of the network over the undirected versions considered in the literature, because in addition to association between disease pairs, the distinction between the statistical properties of the two directions sheds light on which of the two diseases is more probable to cause the other, or at least to precede the other in the causal network that subsumes them both besides other covariates.

Table 17 pertains to disease pairs that have OER>1.5 and are related via shared PPI or genes as deemed by Ref. Park et al. (2009). The table presents the percentage of such disease pairs that are deemed significant by different constructed networks. As expected, OER and *ϕ* have the best performance, and DF is also performing well. These three methods have a local focus, therefore, they are potent in detecting such link-based relations. The other methods, however, focus more on the global structure of the network, and as Table 17 shows, have poor performance for detecting such disease pairs, which matches the expectation.

**Table 17:** Percentage of disease pairs retained by different constructed networks whose gene or PPI commonality are deemed significant by Ref. Park et al. (2009)

	OER	*ϕ*	DF	IPFP	GloSS	Salience
Either direction	68.8	67.2	57.8	1.6	15.6	0
Both directions	43.8	41.1	17.2	1.6	14.1	0
Only one direction	25.0	26.1	40.6	0	1.5	0

### Example application: negative comorbidity and protective effects

As mentioned above, the *ϕ* coefficient and the OER measure can capture negative comorbidities, that is, cases where developing disease A is negatively associated with developing disease B. We can use the *ϕ* correlation to detect strong negative comorbidities, minding that distinguishing actual protection effects from mere negative associations obviously requires more rigorous causal analysis and is not within the scope of this paper. Throughout this paper, we only investigate associations. Here we consider several existing examples in the literature where such negative association has been suggested, and to check if our data replicates these findings. A famous example is the negative association between Alzheimer’s disease and various types of cancer (see Ref. Ma et al. (2014); Tabarés-Seisdedos et al. (2011) and references therein). We focus on the ICD9 codes 140 to 239, which pertain to the neoplasms category, and investigate how the *ϕ* and OER methods perform at capturing negative comorbidities. For the OER method, we look for significant links with OER < 1, and for the *ϕ* method, we look for significant links with *ϕ*<0. The *ϕ* method gives 29 distinct neoplasms exhibiting significant negative comorbidity with Alzheimer’s disease. The OER method detects 26 of those 29 links, and finds no links that the *ϕ* method had not. From these two sets, 27 neoplasms are deemed as significant by both methods. In the converse direction, where the initial diagnosis of a neoplasm is associated with reduced risk of subsequent Alzheimer’s disease, the *ϕ* method detects 30 neoplasms, and the OER detects a subset of them comprising 28 neoplasms. For the Alzheimer’s-neoplasm direction of negative comorbidities, the strongest negative association that the *ϕ* method returns pertains to secondary/unspecified malignant neoplasm of lymph nodes, and for the OER method, the strongest negative association pertains to benign neoplasm of kidney. In the neoplasm-Alzheimer’s direction, the first rank for the *ϕ* method is secondary malignant neoplasm of respiratory and digestive systems, and for the OER method the first rank belongs to malignant neoplasm of gallbladder and extra-hepatic bile ducts.

### Example application: pregnancy-related codes

**Fig. 7:**
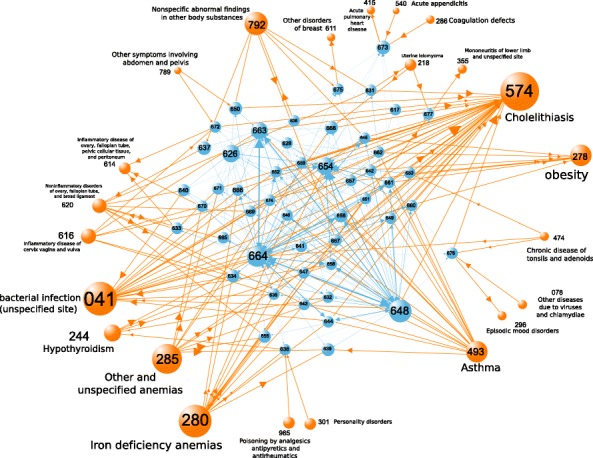
Local network of pregnancy-related codes. The neighborhood of the pregnancy-related codes 630-679 as deemed significant by the *ϕ* method. The blue links are between pregnancy codes and the orange links are between pregnancy and non-pregnancy codes. Nodes sizes are proportional to in-strengths

As discussed above, pregnancy-related codes are among the highly-comorbid codes in several constructed networks. We can use the different constructed networks to investigate these comorbidities. Most codes in the 630-679 range are highly connected to one another. Here we focus on comorbidity with codes outside this category. We consider ICD9 code 634 (spontaneous abortion) as an example. In the OER network, the diseases with the highest correlations that tend to precede 634 include 792 (nonspecific abnormal findings in other body substances), 282 (hereditary hemolytic anemias) and 218 (uterine leiomyoma). These links are also deemed significant by the *ϕ* method. These are in agreement with previous results in the literature (Serjeant et al. 2004; Pajor et al. 1993; Powars et al. 1986; Coronado et al. 2000; Klatsky et al. 2008). The only link OER, *ϕ*, DF, and IPFP methods all agree on is 792. Figure 7 shows the local graph of pregnancy related codes. As stated above, pregnancy-related codes are highly cohesive and each of them connects to many others. For better visualization, we sparsified the neighborhood networks as follows. For each node, we only retained the two pregnancy-related neighbors with highest weights and the two non-pregnancy codes with highest weights. In Fig. 7, the blue links are between pregnancy codes and the orange links are between pregnancy and non-pregnancy codes. We used the *ϕ* network. to generate this graph. Highly-connected nodes in this subnetwork are iron deficiency anemia and other types of anemias (Serjeant et al. 2004; Pajor et al. 1993; Powars et al. 1986) and Cholelithiasis (Ács et al. 2009; Dixon et al. 1987).

### Example application: insight from coarse-grained networks

We now construct a coarse-grained picture of the disease network based on the standard 17 categories of the ICD9 coding scheme (World Health Organization 2004). The list of the 17 categories are presented in Table 18, along with the number of 3-digit ICD9 codes contained within each category, percentage of 3-digit ICD9 codes contained within each category, number of diagnoses in the data set that pertain to diseases within each category, and the percentage of such diagnoses.

**Table 18:** Partitioning the diseases into 17 categories according to the ICD9 codes to obtain a coarse-grained characterization of the disease networks

	Diseases in category	#diseases	%diseases	# prevalence	%prevalence
1	Infectious and parasitic diseases	115	12.57	196992	3.02
2	Neoplasms	93	10.16	376196	5.76
3	Endocrine; nutritional and metabolic diseases; and immunity disorders	39	4.26	763009	11.68
4	Diseases of the blood and blood-forming organs	10	1.09	222671	3.41
5	Mental disorders	30	3.28	308321	4.72
6	Diseases of the nervous system and sense organs	67	7.32	489541	7.50
7	Diseases of the circulatory system	59	6.45	1090435	16.70
8	Diseases of the respiratory system	51	5.57	475937	7.29
9	Diseases of the digestive system	49	5.36	494421	7.57
10	Diseases of the genitourinary system	47	5.14	458440	7.02
11	Complications of pregnancy; childbirth; and the puerperium	49	5.36	436281	6.68
12	Diseases of the skin and subcutaneous tissue	26	2.84	96032	1.47
13	Diseases of the musculoskeletal system and connective tissue	30	3.28	308908	4.73
14	Congenital anomalies	20	2.19	54916	0.84
15	Certain conditions originating in the perinatal period	20	2.19	114847	1.76
16	Symptoms; signs; and ill-defined conditions	20	2.19	331657	5.08
17	Injury and poisoning	190	20.77	311892	4.78

**Table 19:** The percentage of the total link strength of the constructed networks that flows into and out of each of the 17 disease categories in the coarse-grained network

	Raw		OER		phi		DF		IPFP		GloSS		Salience	
	*s*_in_	*s*_out_	*s*_in_	*s*_out_	*s*_in_	*s*_out_	*s*_in_	*s*_out_	*s*_in_	*s*_out_	*s*_in_	*s*_out_	*s*_in_	*s*_out_
1	4.58	3.41	3.60	3.06	4.56	3.03	5.99	3.24	13.04	13.06	5.35	4.42	0.91	1.23
2	3.84	3.74	7.14	7.73	3.43	3.57	1.24	3.39	12.16	12.88	3.81	3.06	0.12	6.28
3	8.32	9.99	3.49	2.73	8.77	10.90	10.46	10.43	2.68	3.13	11.07	13.93	9.37	6.61
4	4.77	4.02	1.58	1.45	4.80	4.06	5.64	4.15	0.21	0.25	8.52	7.05	3.44	2.55
5	4.33	4.86	5.22	5.19	4.53	5.26	3.46	4.71	2.57	2.54	4.00	3.74	1.94	3.49
6	7.03	7.35	9.74	7.26	6.74	7.28	4.07	7.21	5.34	5.07	6.77	5.67	34.99	9.72
7	15.71	19.83	5.19	5.33	17.23	22.68	17.52	21.09	2.80	2.84	17.74	26.35	20.13	19.09
8	8.93	7.59	7.61	5.99	8.96	7.45	9.36	7.71	3.88	4.02	5.32	5.82	1.58	6.27
9	8.94	8.64	6.96	5.66	8.02	7.27	8.31	8.53	2.68	2.68	8.77	6.50	0.11	3.08
10	8.04	7.08	8.23	7.37	8.98	7.23	8.40	7.00	3.87	3.62	3.89	3.33	0.15	4.32
11	2.15	2.70	18.93	20.75	3.11	3.89	2.23	2.76	11.16	10.87	4.86	5.67	24.70	22.37
12	2.38	1.79	3.08	2.25	2.11	1.13	1.97	1.62	1.83	1.92	1.05	0.62	0.00	1.08
13	5.21	5.46	3.84	3.09	4.94	4.82	4.18	5.36	1.50	1.26	4.59	4.10	0.53	5.16
14	0.38	0.52	3.42	3.73	0.24	0.42	0.01	0.40	2.54	2.98	0.08	0.09	0.00	1.15
15	0.06	0.50	0.13	6.33	0.02	0.61	0.00	0.46	6.17	4.89	0.01	0.60	0.45	0.80
16	9.05	6.41	2.80	2.82	8.62	6.07	12.86	6.47	0.58	0.83	8.52	4.21	1.57	0.36
17	6.27	6.11	9.02	9.24	4.93	4.32	4.29	5.46	26.98	27.16	5.66	4.85	0.01	6.43

**Table 20:** The sum of link weight and the percentage of total link weight of the constructed networks that fall within each of the 17 disease categories in the coarse-grained network

	Raw		OER		phi		DF		IPFP		GloSS		Salience	
	#	%	#	%	#	%	#	%	#	%	#	%	#	%
1	34090	0.15	618.04	0.32	21160	0.15	23938	0.17	12.57	4.43	11242	0.21	241	0.18
2	69986	0.31	3726.90	1.93	53246	0.37	37437	0.27	16.32	5.75	23076	0.43	160	0.12
3	185910	0.81	648.26	0.34	145060	1.02	144230	1.03	0.88	0.31	54953	1.03	1100	0.82
4	43323	0.19	162.69	0.08	36460	0.26	37168	0.27	0.04	0.01	35628	0.67	3401	2.55
5	99701	0.44	2224	1.15	92924	0.65	76019	0.55	2.06	0.73	30659	0.58	2134	1.60
6	137860	0.60	3875.10	2.01	95919	0.68	53263	0.38	3.07	1.08	27017	0.51	9392	7.04
7	912650	3.99	2361.10	1.22	799820	5.63	685470	4.91	2.52	0.89	334060	6.27	8869	6.65
8	211480	0.92	2336.60	1.21	187490	1.32	155850	1.12	2.29	0.81	31529	0.59	1333	1.00
9	209060	0.91	1886.30	0.98	142120	1.00	132560	0.95	1.18	0.41	35310	0.66	4	0.00
10	152520	0.67	2982.10	1.54	130710	0.92	105760	0.76	2.47	0.87	20531	0.39	188	0.14
11	305670	1.34	27842	14.41	298570	2.10	276890	1.99	25.06	8.83	229200	4.30	29844	22.37
12	13016	0.06	528.46	0.27	9548	0.07	5360	0.04	0.31	0.11	1457	0.03	0	0.00
13	90585	0.40	988.67	0.51	78006	0.55	54257	0.39	0.28	0.10	18361	0.34	304	0.23
14	3752	0.02	1233.40	0.64	2217	0.02	719	0.01	1.66	0.59	0	0.00	0	0.00
15	3743	0.02	106.01	0.05	296	0.00	455	0.00	10.34	3.64	730	0.01	607	0.45
16	131150	0.57	167.91	0.09	77885	0.55	118540	0.85	0	0.00	13734	0.26	0	0.00
17	111740	0.49	5702.60	2.95	57825	0.41	34586	0.25	47.94	16.89	15082	0.28	17	0.01

The network properties of the 17-node coarse-grained networks are summarized in Table 19, which presents the percentage of the total link weight that flows into and out of each category. Table 20 presents the percentages of the total link weight of the network that falls within each disease category, that is, pertaining to links that connect two nodes that both belong to the same disease category. In the raw network, the highest self-flow belongs to category 7 (disease of the circulatory system), with almost 4% of the total link weight contained inside it. In every method except OER and IPFP, a comparatively high fraction of the total link weight of the network flows within category 7. This category includes, most notably, hypertension, cardiovascular disease, and Ischemic heart disease.

The second highest self-flow in the raw network belongs to category 11 (complications of pregnancy, childbirth, and the puerperium). The OER network changes some of the self-flows markedly. In the OER network, category 11 has an outstandingly high self-flow. Its self-flow is almost the same as the self-flow of every other category combined. So the OER method assigns a high value to disease pairs that both belong to this category. The IPFP method also assigns an outstandingly high self-flow to this category. So, controlling for prevalence, diagnosis pairs that both belong to category 11 typically receive a high weight in the IPFP network. The Salience method assigns the most outstanding self-flow to category 11. In the Salience network, self-flow of category 11 exceeds the self-flows of every other category combined. This means that these diseases are highly central in the disease-disease trajectories, and the links between these diseases contribute to many shortest paths between every disease pair in the whole network.

In Table 21, we present the rankings of the disease categories in terms of what fraction of the total link weight of the network flows out of each category. Except for the IPFP network, the highest out-strength of all networks either belongs to category 11 (complications of pregnancy, childbirth, and the puerperium) or category 7 (disease of the circulatory system). Moreover, category 3 (endocrine/nutritional/metabolic/immunity disorders) is consistently high in out-strength across different networks.

**Table 21:** Ranking of disease categories in terms of out-strength in the constructed networks

Rank	Raw	OER	phi	DF	IPFP	GloSS	Salience
1	7: 15.7%	11: 18.9%	7: 17.2%	7: 17.5%	17: 27.0%	7: 17.7%	11: 35.0%
2	3: 9.1%	17: 9.7%	3: 9.0%	3: 12.9%	1: 13.0%	3: 11.1%	7: 24.7%
3	9: 8.9%	2: 9.0%	8: 9.0%	9: 10.5%	2: 12.2%	4: 8.8%	6: 20.1%
4	8: 8.9%	10: 8.2%	6: 8.8%	8: 9.4%	11: 11.2%	9: 8.5%	3: 9.4%
5	6: 8.3%	6: 7.6%	9: 8.6%	6: 8.4%	6: 6.2%	8: 8.5%	17: 3.4%
6	10: 8.0%	15: 7.1%	10: 8.0%	10: 8.3%	15: 5.3%	11: 6.8%	2: 1.9%
7	16: 7.0%	8: 7.0%	16: 6.7%	16: 6.0%	8: 3.9%	6: 5.7%	8: 1.6%
8	17: 6.3%	9: 5.2%	5: 4.9%	17: 5.6%	10: 3.9%	17: 5.4%	13: 1.6%
9	13: 5.2%	7: 5.2%	13: 4.9%	13: 4.3%	3: 2.8%	1: 5.3%	10: 0.9%
10	5: 4.8%	5: 3.8%	17: 4.8%	5: 4.2%	14: 2.7%	16: 4.9%	5: 0.5%
11	4: 4.6%	14: 3.6%	4: 4.6%	4: 4.1%	7: 2.7%	13: 4.6%	9: 0.5%
12	2: 4.3%	13: 3.5%	11: 4.5%	2: 3.5%	9: 2.6%	5: 4.0%	4: 0.2%
13	1: 3.8%	1: 3.4%	2: 3.4%	1: 2.2%	5: 2.5%	10: 3.9%	1: 0.1%
14	11: 2.4%	16: 3.1%	1: 3.1%	11: 2.0%	12: 1.8%	2: 3.8%	14: 0.1%
15	12: 2.2%	3: 2.8%	12: 2.1%	12: 1.2%	13: 1.5%	12: 1.0%	12: 0.0%
16	14: 0.4%	12: 1.6%	15: 0.2%	15: 0.0%	16: 0.6%	15: 0.1%	15: 0.0%
17	15: 0.1%	4: 0.1%	14: 0.0%	14: 0.0%	4: 0.2%	14: 0.0%	16: 0.0%

Each pair has the form *i*:*x%*, where *i* is the disease category number, and *x* is the percentage of the total link weight of the network that flows out of category *i*

### Example application: comorbidity with the neoplasm category

Many studies in the literature have demonstrated comorbidity patterns between different neoplasms and various other diseases (Mazza and Mitchell 2017; van Baal et al. 2011; Piccirillo et al. 2004; Piccirillo 2000; Fleming et al. 1999; West et al. 1996; Yancik et al. 1996). We can use the coarse-grained networks to investigate neoplasm-related comorbidity patterns. Since we are conditioning on neoplasm being the first diagnosed disease in comorbidity pairs, we can use the DF network. In the coarse-grained DF network, the out-links with the highest weights emanated from the neoplasm category are those to node 7 (diseases of the circulatory system, with 21% of the total link weight of the network), and node 3 (endocrine/nutritional/metabolic/immunity disorders, with 10% of the total link weight). Same is true for the OER and *ϕ* networks; the top two disease categories that follow neoplasms are 7 and 3.

## Conclusion and future work

In this paper we provided a brief summary of some of the main existing methods in the network science literature that could be utilized to construct disease comorbidity networks from longitudinal hospital data. We showed that these methods capture different aspects of the comorbidity patterns, and one must note their properties and choose them according to the structural feature of interest. We presented several examples of the applications of these methods in studying different comorbidity relations, both for single diseases and disease groups. Methodological work in this domain is inchoate, similar to the field of network medicine itself. So there are many interesting unexplored problems of practical significance. Below we highlight a few of such problems.

As discussed above, there are many cases in which a patient visits the hospital and multiple diseases are diagnosed and stored in the data set for the same visit. The temporal direction of such links is lost. We chose to discard such links and refrained from introducing noise to the data set by counting them as bidirectional. An interesting problem would be to infer the direction of these undirected links using *edge recovery* algorithms (Martin et al. 2016) (not to be confused with *link prediction*, where the task is to predict the existence of empirically-absent links). which can also be done as a byproduct of community-detection algorithms (Martin et al. 2016). One has to first devise an inference method which is applicable to weighted directed graphs.

In the construction of the networks discussed in the text, we did not use the covariates, such as age and gender. The interplay between such covariates and structure is worth serious investigation. Manual investigation, such as separating the data for different sexes (such as in Ref. Chmiel et al. (2014); Jensen et al. (2014)) does provide insight, yet a systematic and algorithmic approach would be an interesting research problem. For example, it would be interesting to formulate the macro/meso properties of the comorbidity network, and the local properties of individual diseases, as a function of age. This would be an example of link metadata: a disease would be connected to another disease via multiple links whose metadata (age/sex) are different. In this case, unlike what we did in the present paper, one must not aggregate all the link weights into a single weight. Though methods that incorporate nodal metadata exists (for example, for community detection (Newman and Clauset 2016; Peel et al. 2017)), we are not aware of a systematic investigation for link-based metadata. A simpler approach for the age variable would be to divide the age variables into discrete categories to construct a multiplex network, where each layer represents an age group. Then methods for multiplex network analysis can be applied (e.g., those of community detection (De Bacco et al. 2017)).

Perhaps more important would be to investigate multi-morbidity patterns. This would be the first step towards controlling for age. That is, if instead of comorbidity pairs, we limit the analysis to sets of, say, four diseases, the analysis would differ. We would look for the number of instances that the ordered set of diseases *A*−*B*−*C*−*D* have been diagnosed in the dataset. This probably evinces a more meaningful connection between these diseases, as opposed to only considering pairs separately. In other words, if there are many instances of *A*−*B*−*C*−*D*, this is probably more informative regarding the linkages of these diseases as compared to observing many *A*−*B* and *B*−*C* and *C*−*D* pairs separately. Considering longer chains in such a manner is more likely to capture actual disease progression pathways, because it reduces the likelihood that, for example, *A* simply happens to be a disease that disproportionately occurs in early ages, and *D* at later ages.

Another interesting direction forward would be to incorporate death. One could add death as a new disease. We would obviously have $s_{\text {death}}^{\text {out}}=0$, but it would be instructive to study the in-links and the position of death in the disease network. More particularly, different diseases can be characterized as their distance to this reference node. The first step towards incorporating death into the disease network would be to check the relative network distance of certain diseases, or disease categories, to the death node, which characterizes how deadly those diseases or disease categories are. This also enables focusing the analyses on diseases that tend to appear late, and to characterize their relation to death, and to investigate if they have special properties in this regard.

In addition to death, a more complete analyses would require the addition of a ‘noise’ node, which would represent unknown causes. Because currently, when a disease is diagnosed without any predecessor, it receives no in-link. This limits the analytical power to investigate the importance of unknown causes, which characterizes the likelihood that a healthy person would enter the disease comorbidity network. In other words, the inflow of the network (which represent new patients) all enter through this root node, whence they flow throughout the rest of the network.

Finally, we remark upon the importance of distinguishing between the structural features of acute and chronic diseases in the comorbidity patterns. The temporal profile of comorbidity of disease *A*, if *A* is a chronic disease, looks like: {*A*}→{*A*,*B*}→{*A*,*B*,*C*}→…, because chronic diseases tend to persist, by definition. An alternative pattern could be {*A*}→{*A*,*B*}→{*A*,*C*}→…. On the contrary, if *A* is an acute disease, then the temporal pattern of comorbidities would look like {*A*}→{*B*}→{*C*}→…. These patters presumably undergird different mechanisms of comorbidity, because there is a difference between having a certain disease and having a history of it. Thus a worthwhile problem to study would be the algorithmic characterization of the chronic-ness of diseases and the persistence of their damages, based on their structural properties in the comorbidity network.

## Abbreviations

CMA: Census metropolitan area
DF: Disparity filter
GloSS: Global statistical significance
ICD: International classification of diseases
IPFP: Iterative proportional fitting procedure
OER: Observed-to-expected ratio
PPI: Protein-protein interaction
RR: Relative risk
